# The challenges of institutionalizing community-level social accountability mechanisms for health and nutrition: a qualitative study in Odisha, India

**DOI:** 10.1186/s12913-018-3600-1

**Published:** 2018-10-19

**Authors:** Francesca Feruglio, Nicholas Nisbett

**Affiliations:** Health and Nutrition Research Cluster, Institute of Development Studies at the University of Sussex, Brighton, UK

**Keywords:** Community-level accountability, Marginalized groups and individuals, Maternal and child health, Community service delivery, Empowerment

## Abstract

**Background:**

India has been at the forefront of innovations around social accountability mechanisms in improving the delivery of public services, including health and nutrition. Yet little is known about how such initiatives are faring now that they are incorporated formally into government programmes and implemented at scale. This brings greater impetus to understand their effectiveness. This formative qualitative study focuses on how such mechanisms have sought to strengthen community-level nutrition and health services (the Integrated Child Development Services and the National Rural Health Mission) in the state of Odisha. It fills a gap in the literature on considering how such initiatives are running when institutionalised at scale. The primary research questions were ‘what kinds of community level mechanisms are functioning in randomly selected villages in 3 districts of state of Odisha' and 'how are they perceived to function by their members and frontline workers’.

**Methods:**

The study is based on focus group discussions with pregnant women and mothers of children below the age of 2 (*n* = 12) and with women’s self-help groups (*n* = 12); interviews with frontline health workers (*n* = 24) and with members of community committees (*n* = 36). Interviews were analysed thematically using a priori coding derived from wider literature on key accountability themes.

**Results:**

Four main types of community-based mechanisms were examined – Mothers committees, Jaanch committees, Village Health and Sanitation Committees and Self-Help Groups. The degree of their effectiveness varied depending on their ability to offer meaningful avenues for participation of their members and empower women for autonomous action. Notably, in most of these mechanisms community participation is very weak, with committees largely controlled by the frontline workers who are supposed to be held to account. However, self-help groups showed real levels of autonomy and collective power. Despite not having an explicit accountability role, these groups were nevertheless effective in advocating for better service delivery and the broader needs of their members to a level not seen in institutional committees.

**Conclusions:**

The study points to the need for community-level mechanisms in India to adequately address issues of participation and empowerment of community members to be successful in contributing to service improvements in health and nutrition.

**Electronic supplementary material:**

The online version of this article (10.1186/s12913-018-3600-1) contains supplementary material, which is available to authorized users.

## Background

### Context

India is a middle-income country that performs relatively poorly on health and social development indicators relative to its level of economic growth [1]. Despite being a signatory to a number of international treaties and instruments which promote access to healthcare and nutrition [2], ensuring community level delivery of health and nutrition services remains a major challenge, particularly to disadvantaged populations [3]. Whilst indicators are improving, the progress is slower than it would be desired under ambitious health policy goals. Government funding and investment on health and nutrition services were increasing until recently, though the overall health expenditure is still low at around 1.3% of GDP [4]. However, even with this continued need for further public investment, there is also an argument (shared by those on both sides of the political spectrum) to ensure that governance failures concerning implementation do not also lead to chronic deficiencies in delivery in some of these flagship programmes. These include the Integrated Child Development Services (ICDS) and the National Health Mission (NHM - previously National Rural Health Mission), which are the focus of the paper here (Table 1).

**Table 1:** Background on Village level health and nutrition programmes

The Integrated Child Development Scheme (ICDS) Launched in 1965, ICDS is designed to provide integrated health, nutrition and education services for children from 0 to 6 years of age and for pregnant and lactating mothers. The scheme is overseen nationally by the Ministry for Women and Child Development (WCD). Services are provided through Anganwadi Centres (AWCs), served by honorary but compensated staff members, the Anganwadi worker (AWW) and Anganwadi helper (AWH). Each AWC caters to a population of approximately 1000, with the roles of AWWs and AWHs usually assumed by women appointed within the community. To date, the ICDS structure has concentrated on delivering a package of interventions focused on the following six areas: • Supplementary nutrition; • Growth monitoring; • Maternal and female health counselling; • Immunisation; • Wider health and referral services; and • Pre-school education.	
The National Health Mission Delivery of a wider set of “nutrition-specific” interventions proven to have an impact on maternal and child undernutrition (Bhutta et al. 2013) is dependent on a number of wider health services provided to the community (Avula et al. 2015). This includes services funded and delivered by the Ministry of Health and Family Welfare through the National Health Mission (NHM, formerly National Rural Health Mission), an umbrella scheme launched by the Central Government in 2005 with the objective of reducing the maternal mortality, infant mortality and total fertility rates. The NHM provides for a number of service guarantees free of cost to individuals living below the poverty line (BPL) (NRHM Service Guarantees, GoI). These include services such as: • Antenatal care (ANC) check-ups; • Cash benefits encouraging institutional delivery; • Postpartum visits and IYCF counselling service; • Supplementation of Iron and Folic Acid (IFA) from twelve weeks of pregnancy; and • Prevention and control of childhood diseases like malnutrition. Importantly, the scheme introduced the role of ‘Accredited Social Health Activists’ (ASHAs)^a^ to serve at community-level as the main link between health supply and demand. Similar to AWWs, ASHAs are female honorary volunteers selected from the community and are therefore uniquely placed to provide health and nutritional counselling and services in a complementary manner. Another key figure in the delivery of NHM services is the Auxiliary Nurse Midwife (ANM), whose role is to supervise and guide ASHAs and AWWs in tasks related to the NHM.	

^a^For more details see panel in Balarajan et al. 2011:511

These twin challenges exist in a context of India’s federal system, where health and nutrition programs are designed and (partially) funded at federal level and implemented at state level. Each of India’s 29 States has the flexibility to augment the basic requirements set down by laws and policies developed in Delhi and is also expected to significantly contribute to funding service delivery from State revenues. The context provides room for state-led innovation - seen as critical to effective health delivery, but which remains under-documented in the academic literature [5].

In recognition of the challenges around governance and implementation [6], part of what has been mandated by policy makers at national level [7] is increased monitoring of the delivery of services at community level by community members/service users themselves. This reflects the fact that India has been at the center of social innovation in community monitoring and accountability and social auditing in many sectors [8], where civil society have pioneered a number of community monitoring and accountability systems, some of them now integrated into government programmes [8]. For instance, the ICDS and NHM have undergone efforts to strengthen community participation and oversight by decentralizing service delivery and investing in existing community-level groups and local governance institutions (the latter known collectively as Panchayati Raj Instutions (PRIs) from an older system of Panchayats or village assemblys).

Within this context, this research began as a piece of formative study to support the trial of a community accountability mechanism focused on improving the ICDS and NHM in the Indian State of Odisha, as they are the two main programmes responsible for the delivery of health and nutrition services.

Recent research has recognized Odisha as an innovative state with a strong bureaucracy [9, 10], albeit a state which still suffers significantly from poverty and inequality. Innovation in service delivery has included the implementation of a number of nationally mandated and state-specific initiatives focused on community accountability in relation to the ICDS and NHM. The aim of this research was to scope how such initiatives are actually functioning on the ground and to ascertain the views of service users and frontline workers on their current functioning and on wider governance challenges.

This paper, therefore, is intended to be of benefit to those who want to understand the implementation of such initiatives in the context of large-scale public health systems, and particularly against the backdrop of under-researched innovations occurring in India’s federal structure. As well as documenting important initiatives designed to improve both governance and implementation, evidence on the functioning of community accountability mechanisms at scale helps meet a gap in the development literature which results from the pilot or trial nature of many initiatives to date.

### Concepts and frameworks

This study falls within a wider literature concerning citizens’ participation and social accountability in health service delivery, which has now been covered extensively in several literature reviews [11–15]. A number of studies discuss and evaluate citizen-led action for holding governments accountable for the delivery of public services, including healthcare, housing, social welfare benefits etc. [16, 17]. Within this broad literature, a sub-set of studies focuses on community-based mechanisms that enable citizens to engage with local authorities and service providers, broadly termed ‘social accountability’ and/or ‘transparency and accountability initiatives’ [18, 19].

Community monitoring can be institutionalized and embedded into government programs, or led by stakeholders other than state institutions, such as civil society groups, community based groups and consumer groups. Key means of involving communities in holding service providers accountable include mechanisms such as monitoring committees, citizen juries, scorecards and social audits [16, 20].

Some of the results reported through these initiatives can include increased transparency and participation in decisions concerning service delivery, reduction in corruption practices, improvements in availability and quality of services, and overall changes in power dynamics between citizens and service providers. Crucial to the impact of these initiatives are factors such as the quality of participation of community members [21]; the need for these mechanisms to be ‘vertically integrated’ to address bottlenecks in service delivery at different levels of the supply chain [22, 23]; and the need to trigger sanctions (or the threats of) [24]. At broader level, these types of initiatives should not be seen as technical ‘widgets’ but rather sustained efforts that engage politically with states and service providers [25, 26] and address power dynamics underpinning service delivery, which make a crucial difference in ensuring access to services for marginalized groups such as women and indigenous people. At a local level, effective accountability mechanisms require building inclusive spaces for community participation, whether institutional or informally established, which can reduce ‘asymmetrical power relations’ [14] between local service providers and community members. However if participation is not meaningful, the responsiveness of providers to accountability demands is significantly weaker [15]. For this reason, successful accountability initiatives focus on building awareness, confidence and capacity of community members and ensuring an adequate facilitation of dialogue between citizens and providers [27, 28].

While bearing in mind the broader literature, and concerns about the need for strategic, comprehensive approaches, this study focuses on accountability initiatives operating at a local level, through institutional spaces for participation aimed at improving the delivery of health and nutrition services [7, 29–31]. Factors identified as crucial in the literature - such as the quality of participation within different committees/spaces, the dynamics between citizens and service providers, and actions and impact on service delivery at community level, are thus the primary focus of this exploratory study.

## Methods

The findings of this study are based on primary and secondary data. The field research took place in between December 2015 and January 2016 and consisted of 24 focus group discussion and 60 interviews conducted in twelve villages across the districts of Sundarghar, Khandhamal and Mayurbhanj. The districts were selected as part of a process for a wider trial which followed three purposive criteria: a) population: all districts have a high proportion of ‘Scheduled Tribe’ population, who are poorer than other social groups, have lower access to health services and substantially higher under-five mortality and stunting rates [32]; b) malnutrition rates: one of the districts selected (Sundargarh) has high prevalence of moderate and severe malnutrition (20,27 - 28,29% compared to State average of 17%), and two (Kandhamal and Mayurbhanj) have lower rates of malnutrition (NOP, 2009–13); and c) multiplicity of health and nutrition supply programs and community accountability mechanisms: two of the Districts selected (Kandhamal and Sundargarh) are areas of ‘high-focus’ of intervention for the Government of Odisha [33] to implement additional health and nutritional programs, including community accountability mechanisms to raise awareness and participation in ICDS and NHM services; while one District (Mayurbhanj) is not covered by these additional programs [33]. In each district, villages were stratified between those located close to district headquarters and those located in more remote areas, and two villages from each stratum were selected randomly. The field research was conducted by a team of four skilled qualitative researchers under the supervision of a local survey firm and the lead author.

Secondary data included government documents such as guidelines, informational material and policy documents issued by the Ministry of Health and the Ministry of Women and Child Development. In addition, grey literature on community-level accountability mechanisms was used to shape the following section describing the institutional background of these accountability mechanisms, which is an existing gap in the literature. Tables 2, 3, 4 and 5 describe the participants who were part of the 24 focus group discussions and 60 interviews (hereafter ‘respondents’). These respondents were selected with the help of local frontline workers (the limitations of this are discussed below) on the basis of their role in relevant local groups and their willingness and availability to participate in the study.

**Table 2:** Groups interviewed and methods

Community members entitled to ICDS and NHM services		
Pregnant women and mothers of below 2 years old children	Focus Group Discussions (*n* = 12) (ie 1 per community)	92 individual participants in the FGDs
Frontline health and social workers		
ASHAs	Semi-structured interviews (*n* = 12)	
Anganwadi Workers	Semi-structured interviews (*n* = 12)	
Representatives of community-level groups		
Jaanch Committee	Semi-structured interviews (*n* = 12)	
Mothers Committee	Semi-structured interviews (*n* = 12)	
Gaon Kaliani Samity (GKS)	Semi-structured interviews (*n* = 12)	
Self-Help Groups (SHGs)	Focus Group Discussions (*n* = 12)	109 individual participants in the FGDs

**Table 3:** Education level of respondents - % of respondents

	Committee members	AWWs	ASHAs	Mothers & pregnant mothers
No education	68	0	0	30.5
0-8th Standard	5	36	45.5	27
9–10th Standard	11	55	54.5	31.5
University education	0	0	0	1
No data	16	9	0	10

**Table 4:** Caste composition of respondents - % of respondents

	Committee members	AWWs	ASHAs	Mothers & Pregnant Mothers
SC	43	9	18	48
ST	25	46	46	17
OBC	19	18	9	19
Other	13	9	9	0
No data	0	18	18	16

**Table 5:** Average age of respondents

	Average age
Committee members	38.4
AWWs	42.6
ASHAs	37.7
Mothers and pregnant mothers	24.5

The research tools (Additional file 1, Additional file 2, Additional file 3 and Additional file 4) were designed to elicit the perceptions of service users, frontline workers and committee members on the delivery of health and nutrition services and the role of the committees in ensuring more accountable service delivery. Interviews were semi-structured, focusing on the availability and quality of existing health and nutrition services, the functioning of village-level committees, including the quality of participation and the potential for addressing gaps in service delivery. This format also allowed for un-elicited responses about quality of services or outcomes.

Data analysis was based on narrative and content analysis of the interview transcripts and the secondary data, respectively. The analysis of interviews transcripts paid particular attention to triangulation, by cross-referencing different groups’ answers on broader issues. This allowed consideration of the varying perspectives on the role of frontline workers, the social status of women and the underlying factors that contribute to health and poverty, as well as helping to highlight the underlying biases expressed by different groups of respondents.

One limitation of this study was selection bias related to the role of frontline workers, as part of a wider process of ‘elite capture’ in the village. Research participants were identified by frontline health and nutrition workers,^1^ namely Accredited Social Health Activists (ASHA) and Anganwadi Workers (AWW)^2^ who are likely to have selected among women who are more sympathetic towards their work as well as more aware of their entitlements under health and nutrition schemes. Nonetheless, even given this potential bias, respondents raised a number of concerns with the implementation of health and nutrition schemes, and critical gaps in the role of frontline workers. These form valuable findings reported here.

## Results

### Documenting decentralization and accountability in the delivery of health and nutrition services in India

In India, the two main policies providing for the delivery of health and nutrition services, the ICDS and NHM, have undergone efforts to decentralize service delivery and expand community participation into the governance of public services. To increase accountability and citizen participation, the Government established a number of community-level governance mechanisms, namely committees and groups formed by community members, frontline health and nutrition workers and representatives of local institutions.

The implementation of ICDS services underwent a deep decentralization process following intervention of the Supreme Court of India in 2001, 2004 and 2006. In a landmark judgment on 13th December 2006, the Court ordered the government to ensure “universalisation with quality” and provided measures to decentralize procurement, monitoring and oversight of services at local level, including mandating the role of local women’s Self Help Groups and Mahila Mandals (women’s groups)^3^ [34] in purchasing supplies and preparing and distributing food. In Odisha, local stakeholders were also awarded a major role in monitoring and, to some extent implementing, service delivery through ad-hoc committees: namely Jaanch Committees and Mothers Committees [35].

In Odisha, Self Help Groups (SHGs) are mostly composed of women classified as living “below poverty line” from a specific area [36]. SHGs can be established or run by AWWs, who in most cases have a major role in mobilizing women, but they also may be established independently. The purpose of SHGs is to build women’s economic empowerment and financial independence by obtaining loans and investing it in income generating activities for its members. However, SHGs are also involved in the delivery of health and nutrition services [36], including by procuring and cooking hot meals to be distributed through Anganwadi Centres (see fn. 2).

Jaanch committees [37] (JCs) are formed by five to six members selected among the community and including one retired person, one disabled person, the Mothers’ Committee Chairperson and two members from a local SHG (usually President and Secretary). JCs are tasked with monitoring different aspects of the feeding programs undertaken in AWCs, for instance by overseeing distribution of hot cooked meals and morning snacks which are distributed to women and children enrolled at the AWC.

The Mothers committee (MC) is an all women committee formed by the local ASHAs, a representative from the Panchayat (usually a woman ward member), two members of the SHG, a NGO representative, and one member from each category of beneficiaries: pregnant and lactating women and mothers of children from 6 months to 6 years old selected among community members. MCs have both implementation and monitoring functions. MCs support Anganwadi Workers in the delivery of their services. With regards to their monitoring and oversight role, MCs are tasked with ensuring quality of the food rations distributed at the AWC, including by being present during distribution of take-home rations, and ensuring timely opening of the AWC and home visits by the AWW.

Over a similar period to the universilisation of the ICDS, the National Health Mission (launched in 2005 as the National Rural Health Mission) has been developed to mirror Panchayat Raj Institutions, which are the primary governance mechanisms at community level.^4^

The NHM provides for the establishment of Village Health and Sanitation Committee, (*Gaon Kalyan Samitis,* hereafter GKS) at the village level and Hospital Management Committees attached to lower level health facilities, i.e. Primary Health Centres and Community Health Centres. The GKS are composed of the local ASHA and/or AWW, a representative from the Panchayat (usually a ward member), a representative from the Department of Women and Child Development (usually the Auxiliary Nurse Midwive), two or three representatives from SHGs and a representative from a local NGO.

The primary functions of these committees are planning, implementing and monitoring interventions around health, water and sanitation at village level by developing a health plan based on the village situation and priorities identified by the community. To this end, interventions can range from conducting household surveys, awareness raising on health and WASH, as well as maintenance of the village environment [38]. GKS have an untied annual fund of Rs. 10,000 which can be spent towards activities planned by the Committee. The funding gives GKS more leverage than other village-level committees to improve service delivery. In an effort to increase transparency and accountability of local service delivery, GKS are also meant to collect data on the community and share it with the village council (Gram Sabha) to better coordinate development interventions.

### Community level findings

The findings from the primary research consider the role of community-level mechanisms in making service delivery more responsive to the needs of women and children under 2.

The data collection paid particular attention to services targeted to children under 2 and their mothers, including post-natal care, counseling on infant and young child feeding and supplementary nutrition, given the importance of interventions within the ‘1000 day window of opportunity’ in avoiding mortality and morbidity and lifelong health and developmental consequences [39] .

Results from the interviews conducted with pregnant and lactating women in each village expose gaps in delivering food rations and ensuring access to facility-based and home-based healthcare. Table 6 summarizes key findings emerging from their perceptions and experience. The data confirm numerous challenges in the implementation of ICDS and NRHM which are well-documented in existing research on these two programmes [40–42] and include: experience of inadequate or low quality services; delays and irregularities; discrimination and mistreatment; inaccessibility; and the need for more practicable, and contextually appropriate care and advice. AWWs and ASHAs were also found to be important in accessing and directing services at this local level, with mothers considering them to have agency over processes deemed important, such as the delivery of take home rations and home-based advice. In addition, ASHAs seem to enjoy higher authority and status compared to AWWs, perhaps because of their crucial role in facilitating access to health services within and outside the village.

**Table 6:** Summary of key interview findings - interviews with pregnant women and mothers of children under 2

Type of service used/available	Issues identified by mothers	Relation with FLWs
A. Access to health facilities	Out of pocket expenditures: including for transportation to/from the facility, payments made once at the facility towards health staff or hospital attendants, and at times towards the purchase of equipment and medicine. (existing government policies mandate free transport, treatment and services for women from lower income groups).	ASHAs play an essential role in facilitating access to services and cash entitlements,. This included check-ups and institutional deliveries, which were the services with highest demand among women. In such cases, ASHAs play a key role in arranging transportation by ambulance and, once at the facility, obtaining care promptly. Check-ups and institutional deliveries are also the main requirements for obtaining cash benefits under government schemes designed to encourage demand of health services among below poverty line women
	Respondents faced delay in obtaining care due to referrals from lower-end facilities (such as Primary Health Centers) to higher facilities (such as Community Health Centers and District Level Hospitals). In some cases, referrals take place because of lack of adequate facilities and qualified health staff in lower hospitals.	
	Inadequacy of health facilities: for instance poor hygienic conditions and lack of services which discourage women from seeking institutional care	
	Discrimination on grounds of tribal status: respondents reported being treated poorly by health staff on the basis of their tribal status.	
B. Take home rations	Take home rations are distributed irregularly and the amount is not sufficient: reported waiting time ranged from weeks to three months (in one case). In most cases, availability of eggs was found to be particularly challenging.	The quality of the relation with the AWW generally depends on mothers’ satisfaction with the delivery of take home rations. Although the lack of timely or sufficient distribution of rations may be due to issues beyond the control of AWWs, respondents associated the performance of AWW with the effectiveness and quality of the food received.
	Poor quality: In some cases, rotten food distributed as take home ration caused sickness among women and children.	
	Inaccessibility: women find it difficult to collect the ration from the AWC due to long working hours (AWC distribution ends around 2 pm).	
IYCF Counselling	IYCF advice, through retained by mothers, is not being put into practice. Factors such as poverty, inability to purchase nutritious food, the need to work long hours and in harsh conditions even during pregnancy, prevent them from ensuring adequate nutrition to their children.	Both AWWs and ASHAs are key players in IYCF counselling, which takes place through ad-hoc sessions (Village Health and Nutrition Days) usually held at AWCs. ASHAs also undertake home visits during which they provide IYCF counselling. Home-based care is highly valued by respondents albeit (or precisely because) is not perceived as a duty of ASHAs but rather an ‘extra-mile’ task that she does voluntarily.

Theoretically, accountability mechanisms, including the committees identified here, could play a role in improving the experience of health and nutrition services by helping monitor the work of frontline workers; increasing opportunities for collaboration between the community and workers; identifying structural gaps in the service delivery system and raising awareness of services amongst the community. Against this background, the following section describes the functioning of each of the committees examined, particularly looking at members’ motivations, patterns of participation and actions taken:

#### Jaanch committees

Respondents reported that they had little or no interest in being part of this committee. Primarily, they were selected because they fitted into a required (demographic) category (retired, disabled…) and not because they had a genuine interest in being involved in the Committee. In fact, only a small number were aware of the nature of duties and able/willing to attend to the meetings, as one respondent noted:

*“The AWW madam gives the names of the villagers according to her own choice even the JC members don’t know that they are the members of this committee.” (Jaanch Committee member)*

Confusion about roles and duties of members is also compounded by the fact that some of the respondents were members of multiple committees (e.g. GKS and JC). Among respondents who felt more motivated, the sense of playing an important role in the community was the main factor for becoming a member of the Committee.

Seven respondents were actively engaged in monitoring of food distribution although the nature of their contribution was in question due to the fact that – according to some of them - the process was entirely controlled by the AWW. In fact, oversight was not focused on the AWW but rather on the service users (children or mothers), for instance by ‘making sure that children wash their hands before eating.’ AWWs retain key information about what and how issues should be monitored, and JC members act according to their guidance. In some cases, respondents assisted the AWW in providing the service, such as by helping in cooking and/or purchasing the food. Not all members knew how to contribute, however. Half of the respondents neither had roles to play, nor knew what should be expected from them.^5^

#### Mothers committees

Members were identified and selected by the AWW, so they joined the Committee under the guidance of AWW and were accountable to her. They had a personal stake in ensuring the AWC ran smoothly because they were also users of the AWC. For instance, by being members they learned about the government services available to pregnant and lactating women. As with members of the Jaanch Committees, another motivation for being a member of the Committee was community recognition:

*“Some mothers listen to my information and care for me. That’s why I am feeling happy to work in this sector”.*

Listing the type of action undertaken, members described the committee’s role as serving the community by providing information or helping individuals to access services. Seven respondents reported that the Committee checks the distribution of take-home rations to children and mothers. Six respondents said that the committee counsels mothers on health and nutrition, including counseling around nutrition and feeding practices and facilitating communication between mothers and frontline workers. One respondent mentioned monitoring of home visits. Another four respondents mentioned monitoring the delivery of health services such as Tetanus Toxoid injections, blood pressure tests and vaccinations.^6^

#### Gaon Kaliani Samiti (village health nutrition and sanitation committees)

In line with their mandate, interviewees noted how whilst GKSs were regularly seen to conduct many activities to do with water, sanitation and health, little visible action was taken on nutrition. Estimates were that about one third of the funding received is spent on the committees’ own administration: ASHAs, AWWs and ward members, who are all core members of the GKSs, receive between Rs 50 and Rs 150 for attending GKS meetings, usually convened by the AWW once a month.

Beyond administration, it was reported that GKS funding is allocated towards employing village members to fund small public works, including keeping the village environment clean, repairing tube wells, building platforms in the village wells and cleaning the drainage system, at times conducted in coordination with the Block Development Office (local administration department).

A smaller portion of the funding was reported to be allocated towards promoting healthcare. Here, the role of GKS was two-fold. In most cases the Committee provided financial assistance to individuals in need: for example for purchasing medicines or accessing government hospitals, therefore using the Committee’s funding to overcome shortcomings of the public health system (since under the NHM medicine and ambulance services should be provided free of costs). In a smaller number of cases GKSs organized public awareness activities such as street plays on malaria prevention. However, interviewees unanimously reported that GKS funding is insufficient to carry out (and sustain) the activities required, and that not all GKSs received the disbursement according to the schedule planned.

#### Self help groups

SHGs offer a radically different example of community-level committee based on mutual savings and other collectively beneficial activity. In all villages covered by the study there was more than one SHG (up to six in one case).

Members reported that meetings were held once or twice a month, with the first time for discussing the amount of money that needs to be contributed and the second one to bring a contribution, which is expected of everyone. Attendance at meetings was reported to be very high.

While their main focus is not health and nutrition, SHGs are the only platform, among those examined in the study, to tackle the issues that respondents have identified as (individual and collective) priorities and which are important underlying or basic drivers of health and nutrition outcomes. These included (as mentioned by respondents): poverty and lack of purchasing power, long working hours and heavy workload, gender based violence (often linked to alcohol abuse) and discrimination based on ethnicity and gender.

In addition, in many cases SHGs can play a direct role in facilitating access to services, especially counseling around nutrition and feeding practices. Half of the respondents mentioned the role of SHGs members in providing counseling through home visits, and bridging between pregnant and lactating women and frontline workers. In several other cases SHGs provided cash assistance to pregnant women for medical emergencies, while three respondents mentioned availing of the support of SHGs members to be accompanied to the hospital. In other instances, SHGs played a role in raising awareness on child marriage and nutrition entitlements.

##### AWWs’ perspectives

According to AWWs interviewed, there is a clear distinction between the role of JCs and MCs. While the former plays a stronger oversight role, for instance by ensuring records of food purchase are updated, the latter focuses on supporting the work of the AWW. According to AWWs, both committees help increase demand for services among women, and as a result increase attendance at AWCs for services like vaccination, take home rations and pre-school activities:

*“JC has made my work simpler…. I would have faced problems doing all this but as the members are checking, I am not facing problems... The truth is, those children who did not come to the school… They are coming to school” (AWW)*

In some instances, AWWs noted the important role played by MC members in conveying nutrition and feeding counseling to tribal women, who speak a different language and would not otherwise be able to communicate in Odiya (the mainstream language of the state).

Not all AWWs were satisfied about the work of committees. In many cases the lack of participation in committee meetings was perceived as a hindrance to the functioning of the committee. Lack of attendance was due to little time availability, expectations for incentives (financial or otherwise) and sometimes hindrance exerted by family members (husbands or in-laws), which prevented women from participating.

AWW perspectives on GKS were similar to that noted by others members above. Additionally AWWs noted that funds management has in some cases been cumbersome, as they reported experiencing a significant workload increase for maintaining records and withdrawing the money, which gave rise to conflicts between AWWs and ward members. AWWs also felt being pressurised by community members to allocate the funding towards specific activities.

## Discussion

The results of the research reported here give a helpful picture of how the community monitoring mechanisms and committees mandated under national and state policies are functioning on the ground according to their participants. In considering the constraints to their effective functioning, key themes noted in the conceptual background section, above, appear relevant here – particularly with regards to the quality of participation, the relationships that such initiatives broker between participants and beneficiaries and the ways in which they contribute to a wider sense of empowerment of members. Additional factors raised here include day-to-day practical constraints and incentives to effective participation in the committees studied.

### Functioning of committees

According to relevant government policies, the committees’ mandate relates both to the implementation and monitoring of service delivery. However, both due to the elite capture described and the broader context of under-resourced infrastructure, most of these committees work as extension arms of frontline workers, carrying out tasks and roles that service providers cannot do due to lack of time, capacity and money. The monitoring potential of the committees is thus considerably reduced.

Among the committees examined, the GKS does not exert monitoring functions, but is still seen as very important in the village because of the funding it has access to. Unlike other committees, the impact of the GKS in improving the overall health of the community is perceived as visible and tangible to men and women alike. The strength of the GKS is to fill gaps in service delivery (water, health and sanitation) by funding some of the activities that should be undertaken by the government. In so doing, the GKS acts as a decentralized arm of government, becoming a service provider itself.

JCs showed greater potential for monitoring the performance of AWWs probably because there is a diversity of members including men who are not service recipients, which may help break the provider/users paradigm. However in light of the resource constrains and lack of clarity over the purpose of the committee examined above, in some cases the oversight focuses on service users (children and women) rather than providers (AWWs).

Compared to JCs, MCs are designed to focus more on service delivery than monitoring, and since committee members are all dependent on frontline workers for their access to health and nutrition, their monitoring potential is very limited. In fact, messages delivered by respondents to other service users (women and mothers who are not part of the committee) reproduce the service user-provider dynamic:

*‘We tell them what they should eat and how much rest they should take’ (Respondent)*

#### Quality of participation and nature of the service-provider –beneficiary relationship

The literature on participation highlights the importance of ensuring meaningful participation in accountability initiatives linked to improving service delivery. (21) In the cases considered here, participation was strongly linked with the sense of recognition that motivates people to engage in the committees, the relevance of the committee to the lives of those involved (e.g. mothers participating in the mothers’ committee), and the effectiveness of the committee in improving existing services.

The findings show that most of the committees analysed, albeit described by this paper and by others as ‘community mechanisms’ are in fact institutionally driven and have weak levels of community participation (in terms of numbers and quality of participation). For instance, in GKSs, a heavy presence of members who represent service providers or government institutions (frontline workers, members of PRI and government representatives) leaves little or no space for engagement of community members. In other instances, community members lacked essential information on their role as members and the overall purpose of committees, while in other cases, they were not informed by AWWs about the date and time of the meetings. Frontline workers also maintain official records and deal with financial administration of the committees, effectively playing as gatekeepers.

Quality of participation can also be assessed by looking at inclusion of marginalised individuals in the committees [27]. In the case of JCs, MCs and GKSs bureaucratic and language barriers hindered meaningful participation of marginalized individuals, especially of women with tribal status. For instance, members are required to hold adequate documentation (such as a BPL or Adhaar cards^7^) in order to join MC and JC committees – a requirement often unmet by marginalized women. In the case of committee members from tribal communities, language prevented meaningful engagement during group meetings:

*“There two mothers, who aren’t able to say anything... no good Odia. We do discuss in local language…even if we discuss that in our local language Kandha…then also they just sit silently… They just say yes…whatever we are saying to them, but say nothing.” (GKS member)*

#### Broader practicality constraints and incentives to participation

In the case of MCs and JCs, meetings were poorly attended by community members. AWWs unanimously reported that the poor participation often disrupted the schedule of meetings.

Respondents pointed to the lack of time for attending meetings or carrying out committee-related activities. This is especially true for poorer respondents, who are employed as daily labourers, while most of the respondents who were able to attend the meetings are housewives whose husbands have an occupation. All respondents pointed to the need for rewarding members’ attendance with food or money in line with their wider constraints on time and resources.

Yet time did not seem to be a factor constraining participation to SHG meetings, which were very well attended, even when held twice a month. Such a sharp difference in participation is attributable to the nature of participation in JCs, MCs and GKSs compared to SHGs. Here members join the group voluntarily and with a clear purpose (income earning), which responds to the needs and priorities expressed by women (overcoming poverty). Though in the vast majority of cases SHGs have been established by AWWs, or they hold key positions within it (Secretary or President), the SHGs do not operate as an extension of the work of AWWs, and unlike JCs or MCs, the power within the group is distributed more evenly. As one AWW put it:

*“[The] difference [with] SHG groups is that they are independent. Janch Committee has been selected from the block level. Our villagers have selected the Mothers Committee…. Nobody can tell SHG anything. They do by their own wish.” (AWW, Kamhari Village)*

#### Contributions to empowerment

A study on overall impacts within the broader accountability field has noted the difference between accountability initiatives that lead to service improvements and ones that contribute to broader participation, empowerment and accountability [27]. The more positive responses regarding the functioning of the SHGs reported here show that there are synergies between these two types of outcomes. The power of SHGs appeared to lie in their autonomy and ability to act as a powerful group where women can benefit from peer support as well as gain economic empowerment and confidence [28, 27]. All members have a clear stake in the group, and there is a shared, high commitment to the success of its activities.

The work of SHGs also contributes to women’s empowerment in several ways. Firstly, by increasing financial independence and increased decision-making power over how to spend the money – both individually (earnings) and as a group (loans). Secondly, SHGs effectively create a sense of group and peer-solidarity and become a platform for women to exert their voice and build collective power. This has been essential for achieving important results such as closing down of unlicensed shops selling alcohol (linked to gender based violence). Thirdly, members feel more confident and empowered thanks to the exposure and skills gained by engaging with external stakeholders for business purposes.

SHGs are therefore percieved as a strong platform for women’s empowerment and able to tackle issues that hinder women’s access to health and nutrition services.

#### Service impacts and limits

In the few cases where these mechanisms are effective, the impact seems clear and tangible in the perceptions of their members. When committee members had a genuine interest in the purpose of the committee and elite capture did not undermine opportunities for monitoring and oversight, findings show improvements in both the demand and supply of services.

Respondents highlighted improvements in both service uptake (e.g. more children undertaking preschool education, more women collecting food rations) and service provision (better conditions of AWCs, more timely distribution of food rations and better communications between mothers/women and frontline workers). Some respondents stressed the linkages between the oversight role of committee members, and accountable and responsive AWWs:

*“They are unable to hide things as we work honestly and looking around every day (…) this is possible because we came together”* (MC member, Rayapalli Village)

However, many of the gaps in the delivery of health and nutrition services that were raised during group discussions with mothers may fall much beyond the control of frontline workers. For instance, unavailability of food stocks in AWCs results from bottlenecks within the Food and Civil Supplies Department rather than on the performance of specific AWWs. Similarly, the poor availability of ambulances and quality healthcare in government hospitals are infrastructural issues for which the Department for Health and Family Welfare is responsible. In cases when frontline workers are unable or unwilling to respond to grievances raised by service users, these are left with no other channel to refer their grievance to. These limitations highlight the need for community committees to be integrated with higher-level grievance mechanisms able to address systemic issues.

## Conclusions

Community-based monitoring aims to ensure a more responsive and accountable delivery of essential services by involving service users in the monitoring, oversight and planning of those services. In India, community monitoring mechanisms have been established within wider decentralization efforts undertaken by the Government. The two main policies providing for the delivery of health and nutrition services, NHM and ICDS, envisage a number of community-based mechanisms formed by community members, frontline health and nutrition workers and representatives of local institutions which are tasked to ensure effective and responsive delivery of health and nutrition entitlements, particularly to women and children.

This study sought to understand the effectiveness of such community-level mechanisms in the State of Odisha, India.

Findings highlighted how in these institutionalized mechanisms the participation of community members is often tokenistic, as committees are effectively controlled by the frontline workers who are supposed to be held into account. The cases examined reveal a significant degree of confusion between accountability and decentralization functions, which these committees are both invested with. The confusion stems from a lack of meaningful participation of community members, who are mostly not informed of the committees’ functions and decision-making processes, and end up acting as extension arms of (often overworked) frontline workers. In the few instances where these committees have been able to oversee the performance of frontline workers, with reported improvements in service delivery, they have been unable to address wider, systemic issues along the supply chain of health and nutrition services.

SHGs appear to stand out from the rest of the committees analysed. Important lessons can be drawn from SHGs with regards to meaningful participation and building of collective power [28], which are relevant to the effectiveness of accountability mechanisms. The autonomy of the committee, members’ strong commitment to the success of the group, and a common goal are among its key strengths, along with the ability to respond to real needs and priorities of women living in villages. SHGs don’t have a specific mandate for monitoring and overseeing service delivery, nonetheless they were reported as playing a key role in addressing issues women face, including lack of access to services, and building awareness and agency of women. In addition, they act as a pressure group in defending women’s interests and changing power dynamics at family and community levels. Future efforts may do well to either build on these types of groups; or incorporate their structural advantages, rather than build new, institutionalized mechanisms afresh.

## Additional files

Additional file 1: Focus Group Discussion schedule – mothers. (PDF 678 kb)

Additional file 2: Focus Group Discussion schedule – SHG members. (PDF 614 kb)

Additional file 3: In-Depth Interview Schedule - ASHA or AWW. (PDF 596 kb)

Additional file 4: In-Depth Interview Schedule – Committee Members. (PDF 497 kb)

## Abbreviations

ASHA: Accredited Social Health Activist
AWC: *Anganwadi* Centre
AWW: *Anganwadi* Worker
BPL: Below Poverty Line
GDP: Gross Domestic Product
GKS: *Gaon Kalyan Samitis* (Village Health and Sanitaiton Committees)
ICDS: Integrated Child Development Services
JC: *Jaanch* Committee
MC: Mothers’ Committee
NHM: National Health Mission
NRHM: National Rural Health Mission
PRI: *Panchayati Raj* Institution
SHG: Self Help Groups
WASH: Water Sanitation and Hygiene

## Footnotes

1 Government workers are often ‘gatekeepers’ in such contexts and possess the access to and knowledge of committee/beneficiary lists that may be possible to bypass in a more prolonged study, but not in a rapidly executed formative study.

2 ASHAs are the backbone of the National Rural Health Mission. They are the primary point of contact for pregnant and lactating women and children to avail of benefits and entitlements guaranteed under the NRHM. ASHAs operate at village level and often they are selected within the village they work in. This leads to strong ties between ASHAs and women. AWWs, instead, are in charge of delivering services mandated under the Integrated Child Development Scheme (ICDS) which include food rations and take-home rations, educational services for children under 6 and measures to identify and contrast malnutrition. AWWs operate at village level through Anganwadi Centres (AWCs).

3 The Court instructed that “… preferably ICDS funds shall be spent by making use of village communities, self-help groups and Mahila Mandals (Women’s groups) for buying of grains and preparation of meals; Local women’s Self Help Groups (SHGs) and Mahila Mandals [Women’s Groups] should be encouraged to supply the supplementary food distributed in Anganwadis centers. They can make purchases, prepare the food locally, and supervise the distribution.”

4 NHM/NRHM monitoring efforts have been led at institutional level by the Advisory Group on Community Action. This is a standing group established by the Ministry of Health which comprises public health professionals associated with major NGOs and meets regularly to guide activities relating to community action. AGCA coordinated the first phase of Community Monitoring in nine States of the country between 2007 and 2009 by coordinating grassroots efforts with various groups such as community members and beneficiaries, community-based organizations and NGOs working with communities, along with panchayat representatives.

5 To increase the members’ participation and engagement, the following suggestions were raised: a) providing meals for members (four respondents); b) ensuring the AWW provides clear information on roles and responsibilities of Committee members (three respondents); c) allowing mothers with infants to attend the meetings; d) arranging meetings with mothers (two respondents) and e) provide the opportunity for those who are genuinely interested in serving on the committee to replace members who are not motivated (one respondent).

6 TT shots, blood pressure tests and vaccinations are some of the main services pregnant and lactating women are entitled to under the National Rural Health Mission. These are meant to be provided free of cost during pregnancy and after delivery.

7 Types of identification cards often required to access services and benefits.
