# Supplementary material for: The challenges of institutionalizing community-level social accountability mechanisms for health and nutrition: a qualitative study in Odisha, India
Source: BMC Health Serv Res. 2018 Oct 19;18:788. doi: 10.1186/s12913-018-3600-1 (PMC6194642; doi:10.1186/s12913-018-3600-1)
Supplement: Supplementary file 1 — Focus Group Discussion schedule – mothers. (PDF 678 kb) [file 12913_2018_3600_MOESM1_ESM.pdf]

DRAFT – FGD with mothers with children below 2 years of age

Interview code: ☐ Audio File code: ☐

Date:

District: Block:

Village: Location of interview:

Interviewer Name: Note Taker Names:

CONSENT OF RESPONDENT

<u>ଉତ୍ତରଦାତାଙ୍କ ସମ୍ମତି ପ୍ରଦାନ</u>

Thank you for this opportunity to speak with you. Together with the Institute for Development Studies (IDS), we are conducting a survey that will provide us with necessary information to carry out research that is designed to help promote the welfare of people in Odisha; particularly, to improve food consumption and nutrition of the people, and to enhance community accountability and income generation.

ଆପଣଙ୍କ ସହିତ ଆଲୋଚନା କରିବା ପାଇଁ ସୁଯୋଗ ମିଳିଥିବାରୁ ଧନ୍ୟବାଦ ଜଣାଉଛି । ଆମେ institute of development studies (IDS) ସହିତ ଏକ ସର୍ଭେ କାର୍ଯ୍ୟ କରୁଛୁ ଏଥିରୁ ଯେଉଁ ସୁଚନା ସବୁ ମିଳିବ ତାହାକୁ ଆଧାର କରି ଆମ୍ଭେ ଏକ ଅନୁଧ୍ୟାନ ବା research କରିବୁ ଯେଉଁଥିରେ କି ଓଡିଶା ବାସିଙ୍କ ମଗଂଳ ବିଶେଷ କରି ସମାନକଂର ଖାଦ୍ୟା ଖାଇବା ଏବଂ ଲୋକମାନକଂର ପୌଷ୍ଟିକ ସ୍ଥିତି ତଥା ଆୟ ପନ୍ଥା ସୃଷ୍ଟି ଏବଂ ଗୋଷ୍ଟି ଉତ୍ତର ଦାଇତ୍ବ ସ୍ଥାନ ପାଇବ ।

We are inviting you to be a participant in this study. We value your opinion and there are no wrong answers to the questions we will be asking in the interview. We will use approximately 45 minutes of your time to collect all the information. There will be no cost to you other than your time. There will be no risk as a result of your participating in the study. Your participation in this research is completely voluntary. You are free to withdraw your consent and discontinue participation in this study at any time.

This study is conducted anonymously. You will only be identified through code numbers. Your identity will not be stored with other information we collect about you. Any information we obtain from you during the research will be kept strictly confidential. This interview will be audio recorded and its content will not be shared or used outside the scope of this research.

ଏହି ଅନୁଧ୍ୟାନରେ ଏକ ଅଂଶ ଗ୍ରହଣ କରି ହେବା ପାଇଁ ଆମେ ଆପଣଙ୍କୁ ଅନୁରୋଧ କରୁଛୁ ଆମେ ପଚାରିବା ଯେ କୌଣସି ପ୍ରଶ୍ନ ପାଇଁ ଆପଣଙ୍କ ମତାମତ କୁ ଆମେ ଗୁରତ୍ବର ସହ ଗ୍ରହଣ କରିବୁ ଆଉ ଏକ କଥା କେଉଛି ଏଥିରେ କୌଣସି ଉତ୍ତର ଭୁଲ୍ ଠିକ୍ ର ବିବେଚନା ମଧ କରଜିବ ନାହିଁ । କଥାଟି ହେଲା ଆପଣ କେବଳ ଏଥିପାଇଁ ସମୟ ଦେବା ବ୍ୟତିତ ଆପଣଙ୍କୁ ଆଉ କିଛି ଦବାର ଆବସ୍ୟାକତା ନାହିଁ । ସୁଚନା ସଂଗ୍ରହ ନିମନ୍ତେ ଆମ୍ଭେ ଆପଣଙ୍କର ୪୫ ମିନିଟ୍ ସମୟ ନେବୁ ଏହି ଅନୁଧ୍ୟାନର ଆପଣଙ୍କ ଅଂଶ ଗ୍ରହଣ ସମ୍ପୁର୍ଣ ଇଛାଧିନ । ଏହି ଅନୁଧ୍ୟାନରେ ଅଂଶ ଗ୍ରହଣ କରିବାର ସମ୍ମତି କୁ ଆପଣ ଯେ କୌଣସି ସମୟରେ ଫେରାଇ ଦେଇ ଅନୁଧ୍ୟାନ ଅଂଶ ଗ୍ରହଣ କରିବୁ ମନା କରିଦେଇ ପାରନ୍ତି । ଅନୁଧ୍ୟାନ ଟିରେ ସମ୍ପୁର୍ଣ ଗୋପନୀୟତା ଭାଷା କରା ଯାଇଛି ଏଥିରେ ଆପଣଙ୍କ ପରିଚୟ ଏକ କୋଡ୍ ନମ୍ବର୍ ହିଁ ରହିବ ଅନ୍ୟ କୌଣସି ସୁଚନା ସହିତ ଆପଣଙ୍କ ପରିଚୟ କୁ ସଲଗ୍ନ କରାଯିବ ନାହିଁ ଅନୁଧ୍ୟାନ ପ୍ରକ୍ରିୟା ରେ ଆପଣଙ୍କ ଠାରୁ ସମ୍ପୁର୍ଣ ଗୋପନ ରଖାଯିବ । ଏହି ସାକ୍ଷାତକାରଟିକୁ ଆମେ ରେକର୍ଡ କରିବାକୁ ଚାହୁଁଚୁ ଏହାକୁ କେବଳ ଆମେ ରିସର୍ଚ୍ ପାଇଁ ବ୍ୟବହାର କରିବୁ , ଅନ୍ୟ କେଉଁଠି ପ୍ରକାଶ କରାଯିବ ନାହିଁ..

Your participation will be highly appreciated. The answers you give will help provide better information to policy-makers, practitioners and program managers so that they can plan for better services that will respond to your needs.

ଅନୁଧ୍ୟାନରେ ଆପଣଙ୍କ ଅଂସ ଗ୍ରହଣ ପ୍ରଶଂଶନିୟ ହୋଇ ରହିବ । ଅନୁଧ୍ୟାନ ମାଧ୍ୟମରେ ଆପଣଙ୍କ ଉତ୍ତର ଓ ସୁଚନା ଗୁଡିକ ନୀତି ନିୟମ ପ୍ରସ୍ତୁତ କରି, କାର୍ଯ୍ୟକାରୀ ପେଶାଦାର, କାର୍ଯ୍ୟକ୍ରମ ପରିଚାଳନା କାରିକୁ ଖୁରାକ ଯୋଗାଇବା, ଫଳତଃ ସେମାନେ ଆପଣଙ୍କ ଉତ୍ତର ମାଧ୍ୟମରେ ଉପସ୍ଥାପନ କରିଥିବା ଆବଶ୍ୟକତାର ପୁରଣ ନିମନ୍ତେ ଓ ସେବା ଯୋଗାଣ ନିମନ୍ତେ ଉତ୍ତମ ଯୋଜନା ପ୍ରସ୍ତୁତ କରିବାରେ ଉତ୍ତମ ଯୋଜନା ଅରିପାରିବେ ।

The researcher read to me orally the consent form and explained to me its meaning. I agree to take part in this research. I understand that I am free to discontinue participation at any time if I so choose, and that the investigator will gladly answer any question that arise during the course of the research.

ଅନୁଧ୍ୟାନକାରି ସମ୍ମତି ପତ୍ର କୁ ମୋ ସାମ୍ନାରେ ସମ୍ପୁର୍ଣ ଭାବେ ପଢ଼ି ସଣାଇ ଛନ୍ତି ଏବଂ ଏହାର ଅର୍ଥ ମତେ ବୁଝାଇ ଛନ୍ତି ଏହି ଅନୁଧ୍ୟାନ ରେ ଭାଗ ନେବା ପାଇଁ ମୁ ରାଜି । ମୁ ଭଲଭାବେ ଜନିଛି ଯେ ଯେକୌଣସି ସମୟରେ ମୁ ଅନୁଧ୍ୟାନର ନିଜକୁ ହେରାଇ ଆଣିପାରିବ ଏବଂ ଅନୁଧ୍ୟାନକାରି ଇଣ୍ଟରଭ୍ୟୁ ମଧ୍ୟରେ ଉପୁଜିଥିବା ପ୍ରଶ୍ନ ଗୁଡିକ ର ଉତ୍ତର ଖୁସିରେ ଦେବେ ।

Contact Persons:

ଯୋଗାଯୋଗ ଠିକଣା

Satyanarayan Mohanty, DCOR Consulting

Address: DCOR Consulting Pvt. Ltd., 131 (P), Punjabi Chhak, Satyanagar, Odisha, India, Pin – 751007

Tel: +91-9437698965, E-mail: satya.dcor@gmail.com

Dr. Nicholas Nisbett, IDS

Address: Institute of Development Studies, University of Sussex, Brighton BN1 9RE

Tel: +44 (0)1273 606261; E-mail: n.nisbett@ids.ac.uk

Please tick mark on the right box depending on the respondent's consent

ଉତ୍ତର ଦାତା/ଦାତ୍ରୀ ସମ୍ମତିକୁ ଭିତି କରି ନିରଧିଷ୍ଟ କୋଠରୀରେ ଠିକ୍ ଚିହ୍ନ ଦିଅନ୍ତୁ

Consent given: ସମ୍ମତି ପ୍ରଦାନ	Yes	No

Signature of the Enumerator: ______________________   **Date: DD/_______/_______/_______/**

A. FGD description: ଉଦ୍ଧିଷ୍ଟ ଦଳଗତ ଆଲୋଚନାର ବର୍ଣ୍ଣନା

1. No. of people attending FGD: ଉଦ୍ଧିଷ୍ଟ ଦଳଗତ ଆଲୋଚନାରେ ଯୋଗଦେଇଥିବା ଲୋକମାନଂକ ସଂଖ୍ୟା /Ethnicity: ଜାତି/ମୌଳିକ ସମ୍ପ୍ରଦାୟ

3. Religion: ଧର୍ମ

4. Education: ଶିକ୍ଷା

	Hamlet Name	Age of Mother (in completed years)	Age of Index Child (in completed months)	Caste/ Ethnicity	Religion	Education
Mother 1						
Mother 2						
Mother 3						
Mother 4						
Mother 5						
Mother 6						
Mother 7						
Mother 8						

B. Awareness and perception of health services
ସଚେତନତା ଏବଂ ସ୍ବାସ୍ଥ୍ୟସେବା ବିଷୟକ ଧାରଣା

1. Could you please describe your own overall experience with Government health services outside your village (for instance Sub-centre, PHC, CHC – no ASHA and AWW) during your last pregnancy.
ଆପଣକର ଶେଷ ଗର୍ଭ ସମୟରେ ସରକାରୀ ସ୍ବାସ୍ଥ୍ୟସେବା ବାବଦରେ ଅଭିଜ୍ଞତା କହିବେକି?

Prompts: What type of services you received?
ସୂଚାଇ କୁହନ୍ତୁ: ଆପଣ କେଉଁ ପ୍ରକାର ସେବା ପାଇଥିଲେ?

What problems did you face? Please provide examples (For instance, I had to spend a lot of money, I felt I did not receive adequate care, the hospital was not clean etc.)
ଆପଣ କେଉଁସବୁ ସମସ୍ୟାର ସମ୍ମୁଖିନ ହୋଇଥିଲେ? ଉଦାହରଣ ସ୍ବରୁପ, ମତେ ଅନେକ ଟଂକା ଖର୍ଚ କରିବାକୁ ପଡିଥିଲା, ମତେ ଲାଗିଲା ଯେ ମୁ ଯଥେଷ୍ଟ ସେବା ପାଇ ପାରିଲିନୀ, ଡାକ୍ତରଖାନା ପରିସ୍କାର ପରିଛନ୍ନ ନଥିଲା ଇତ୍ୟଦି!
Were you satisfied with them? What else you would have needed?
ଆପଣ ସେମାନଂକ ଉପରେ ସନ୍ତୁଷ୍ଟ ଥିଲେ କି? ସେଠାରେ ଆପଣ ଆଉ କେଉସବୁ ଆବସ୍ୟକତା ମେଣ୍ଟା!ଇ ପାରିଥାନ୍ତେ ବୋଲି ଆପଣ ଭାବୁଛନ୍ତି?

2. In your knowledge, what are some of the health services you are entitled to receive at home and in your village during pregnancy?
ଆପଣଙ୍କ ଜାଣିବାରେ ଆପଣ ନିଜ ଘରେ ଓ ଗ୍ରାମରେ କେଉଁସବୁ ସ୍ୱାସ୍ଥ୍ୟସେବା ପାଇବାର ହକିଦାର୍?(ଗର୍ଭବତୀ ଥିବା ସମୟରେ)
Prompt the followings, allowing time for people to say what they know for each of these: Food supplements, Cash assistance, Counselling on feeding practices. Any other service?
ନିମ୍ନଗୁଡିକୁ ସୁଚାଇ କୁହନ୍ତୁ: ସେମାନଙ୍କୁ ସମୟ ଦେଇ ପଚାରନ୍ତୁ ଯେ ସେମାନେ ଏଗୁଡିକ ବାବଦରେ କଣ ସବୁ ଜାଣିଛନ୍ତି? ଅତିରିକ୍ତ ଖାଦ୍ୟ ଯୋଗାଣ, ଆର୍ଥିକ ସହାୟତା ସ୍ତନ୍ୟପାନ ଅଭ୍ୟାସ ବାବଦରେ ପରାମର୍ଶ ଦେବା ଅନ୍ୟାନ୍ୟ ସେବା ସମୁହ?

3. What has been your experience with these services (services mentioned in Q2) ? Have you received them? If yes, were you satisfied with the quality?
If no, why do you think you did not receive these services? Probe: Was it an issue related to unavailability of services? Economic barriers (fees)? Caste or socioeconomic background?
ଏହି ସେବା ବାବଦରେ ଆପଣଂକର କି ପ୍ରକାରର ଅଭିଜ୍ଞତା ରହିଚି?ଆପଣ ଏହି ସେବା ଗୁଡିକ ପାଇଛନ୍ତି କି ?ଯଦି ପାଇଥିଲେ ସେବାର ଗୁଣାତ୍ମକ ମାନ ସନ୍ତୋଷ ଜନକ ଥିଲା କି?ଯଦି ନୁହଁ ଆପଣଂକୁ ଏହି ସବୁ କାହିକି ମିଳିନଥିଲା ବୋଲି ଭାବୁଛନ୍ତି?ସମସ୍ୟାଟି ସେବା ଉପଲବ୍ଧ ନ ଥିବା ଜନିତ ଥିଲା କି? ଅର୍ଥାଭାବ ଜନିତ କାରଣ ଯଥା ଫିସ୍ ଇତ୍ୟାଦି ଥିଲା କି? ଜାତିଭେଦ ବା ଅର୍ଥିକ ଓ ସାମାଜିକ ଜନିତ କାରଣ ଥିଲା କି?

<u>B. Relation with FLWs (କ୍ଷେତ୍ର କର୍ମିଂକ ସହିତ ସମ୍ପର୍କ)</u>

1. What’s your relation with the local ASHA like?
ସ୍ଥାନୀୟ ଆଶା ସହିତ ଆପଣଂକ ସମ୍ପର୍କ କିପରି?
Prompts: Does she reach out to you or do you approach her?
ସୂଚାଇ କୁହନ୍ତୁ: ଯେପରିକି ସେ ଆପଣଂକ ପାଖକୁ ଆସନ୍ତି ବା ଆପଣ ବି ତାଂକ ପାଖକୁ ଯାନ୍ତି?
Do you feel she is helpful? In what way?
ଆପଣ ଭାବୁଚନ୍ତି କି ସେ ଆପଣଂକର ବହୁ ସମୟରେ କାମରେ ଆସନ୍ତି? କେଉଁ ଭଳି ଭାବରେ?
2. What’s your relation with the local AWW like?
ସ୍ଥାନୀୟ AWW ସହିତ ଆପଣଂକର ସମ୍ପର୍କ କିପରି?
Prompts: Do you visit the AWC often?
ସୂଚାଇ କୁହନ୍ତୁ: ଆପଣ କେବେକେବେ AWC କୁ ଯାଇଥାନ୍ତି ?
What kind of services do you find? Do you find them useful?
ସେଠାରେ କଣ ସବୁ ମିଳୁ ଥିବାର ଆପଣ ଜାଣନ୍ତି?ସେ ସେବା ଗୁଡିକ ଆପଣଂକୁ ପାଇଁ ଦରକାର କି ?
What could be improved?
ଏଥିରେ କି ପ୍ରକାର ଉନ୍ନତି ଆଣିବା ଉଚିତ୍?
3. Can you give us an example of the support received by the ASHA or AWW during your last pregnancy? Was it useful?
ଗତ ଗର୍ଭଧାରଣ ସମୟରେ ଆପଣ ଆଶା କିମ୍ବା AWW କର୍ମିଂକ ଠାରୁ କୌଣସି ସହଯୋଗ ପାଇଥିଲେ କି? ଏହା ଆପଣଂକ ପାଇ ଉପୋଯୋଗୀ ଥିଲା କି?

Prompts: What was the support about/how did she help? What did she tell you? ସୁଚାଇ କୁହନ୍ତୁ: କେଉଁ ପ୍ରକାରର ସହାୟତା ଥିଲା ! ସେ କିପରି ଭାବେ ସହଯୋଗ କଲେ? ସେ ଆପଣଂକୁ କଣ କହିଲେ?

4. What else you would have needed? What suggestions do you have to improve these services? ଆପଣ ଆଉ କଣ ଆବଶ୍ୟକ କରୁଥିଲେ? ଆପଣଂକ ମତରେ ଏହି ସେବା ଗୁଡିକରେ କିପରି ଉନତି ଆସିପାରିବ?

5. Have you received counselling or advising on nutrition, such as supplementary nutrition during your pregnancy and advising on how and what to feed your child? Who and where counselled you on how to feed your child (AWC, VHSND)?

ଆପଣଂକୁ ଗର୍ଭ ସମୟରେ ପୃଷ୍ଟି ବାବଦରେ ବିଶେଷ କରି ଅନୁରୁପ ଖାଦ୍ୟ (Supplementary Nutrition) କିଛି ପରାମର୍ଶ ଦିଆ ଯାଇଛି ଏବଂଶିଶୁ କଣ ଖାଇବ ଓ କିପରି ଏ ବାବଦରେ ଉପଦେଶ ଦିଆଯାଇଛି କି? ଶିଶୁ କୁ କିପରି ଖୁଆଇବାକୁ ହେବ ସେ ବିଷୟରେ କିଏ ଏବଂ କେଉଁଠାରେ କହିଥିଲେ?

Prompt: What kind of things were you told?

ସୁଚାଇ କୁହନ୍ତୁ: ସେଠାରେ ଆପାଣ କେଉଁ ବିଷୟରେ କହିଥାନ୍ତି ? (AWC, VHSND)?

ସେମାନେ ଯାହା କହିଥାନ୍ତି ଆପଣ ଗ୍ରହଣ କରିଥାନ୍ତି ? ଯଦି ନୁହେ କାହିଁକି (ଉଦାହରଣ : ଖାଦ୍ୟର ଅଭାବ,ସମୟର ଅଭାବ)

7. During your last pregnancy, have you been given a Mothers and Child Protection card? Who gave it to you and kept it updated? Have you found it a useful tool to receive practical advise on your pregnancy and child health?(e.g. not in my language/cannot read/not been explained how to use it etc…)

ଶେଷ ଗର୍ଭ ସମୟରେ ତୁମକୁ ମା ସିଶୁ ସୁରକ୍ଷା କାର୍ଡ଼ (ମମତା କାର୍ଡ଼)ମିଳିଥିଲା କି? ଏହି କାର୍ଡ଼ କୁ କିଏ ଦେଇଥିଲେ ଏବଂ ଏହି କାର୍ଡ଼ କୁ କିଏ ନିୟମିତ ଲେଖୁଥିଲେ? ଆପଣଂକ ଗର୍ଭାବସ୍ଥା ଏବଂ ଶିଶୁ ସ୍ବାସ୍ଥ୍ୟ ନିମନ୍ତେ କାର୍ଡ଼ ଟି ଆପଣଂକ ପାଇଁ ପ୍ରକୃତ ପକ୍ଷେ ଏକ ଉପଦେଶ ମୁଳକ ତଥା ଉପୋଯୋଗୀ ସାଧନ ଭାବେ ରହିଥିଲା କି?

(ଉଦାହରଣ ସ୍ବରୁପ: ମୋ ଭାଷାରେ ନଥିଲା/ପଢ଼ି ପରୁନଥିଲି ଏହାକୁ କିପରି ବ୍ୟବହାର କରିବାକୁ ହେବ ମତେ ବୁଝାଇ ଦିଆଯାଇନଥିଲା?)

C. Existing community-level resources: (ରହିଥିବା ଗୋଷ୍ଠୀ ଭିତିକ ସମ୍ବଳ)

1. Suppose you have an issue with the ASHA/AWW: who would you seek advice from?
ଆଶା ଏବଂ AWW କର୍ମୀ କୁ ନେଇ ବାହାରେ କିଛି ସମସ୍ୟା ଦେଖା ଦେଇଥିବାର ଆପଣଂକ ନଜରକୁ ଆସିଛି କି?

2. Suppose you have an issue that is not health-related (for instance problems in the family, or at the workplace): Who do you approach/seek advice from?
ସ୍ବାସ୍ଥ୍ୟ ବ୍ୟତିତ କାହାର ଅନ୍ୟ କୌଣସି ସମସ୍ୟା ପରିବାରରେ କିମ୍ବା କାର୍ଯ୍ୟ କ୍ଷେତ୍ରରେ ସେପରି କିଛି ଆପଣଂକ ନଜରରେ ଅଛି କି? କିଏ ଆପଣମାନକଂ ପାଖକୁ ଯାଇ ସ୍ବାସ୍ଥ୍ୟ ବିଷୟରେ କୁହନ୍ତି ?

3. What would you say are the main issues *you* face *as women in your community*?
ଜଣେ ମହିଳା ଭାବେ ଆପଣ କେଉଁ ଭଳି ସମସ୍ୟା ର ସମ୍ମୁଖିନ ହେଉଛନ୍ତି ବୋଲି କୁହନ୍ତୁ?

4. What do you think are the main causes of these issues? And what do you feel could be done to address them?
ଏହି ସମସ୍ୟା ଗୁଡିକର ମୁଳ କାରଣ ଗୁଡିକ କଣ ବୋଲି ଆପଣ ଭାବୁଛନ୍ତି? ଏବଂ ସମସ୍ୟା ସମାଧାନ ପାଇଁ କଣ ଦରକାର ବୋଲି ଭାବୁଛନ୍ତି?

5. Do you feel that women from other communities face the same issues you do? What's the difference?
ଆପଣ ଭାବୁଛନ୍ତି କି ଅନ୍ୟ ମହିଳା ମାନେ ଆପଣଂକ ଭଳି ସମସ୍ୟା ର ସମୁଖିନ ହେଉଥିବେ? ଏଥିରେ ବ୍ୟବଧାନ କେତେ ଦେଖୁଛନ୍ତି ?

6. In your village, are there groups or institutions, or individuals, who address/work towards addressing women's problems? In what way are they helpful/What do they do? *If difficulty in recalling, prompt on existing community-level institutions/groups such as SHG, Jaanch C, Mothers' C, GKS etc.*
ମହିଳା ମାନଙ୍କର ସମସ୍ୟାର ସମାଧାନ ନିମନ୍ତେ ଆପଣଂକ ଗ୍ରାମରେ ସେମିତି କିଛି ଅନୁଷ୍ଠାନ/ଦଳ/ବ୍ୟକ୍ତି ବିଶେଷ ରହିଥିବାର ଆପଣଂକୁ ଜଣା ଅଛି କି? କେଉଁ ଦିଗରୁ ସେମାନେ ସହାୟକ ଅଛନ୍ତି ଏବଂ ସେମାନେ କଣ କରିଥାନ୍ତି?
(ଏହା ମନେ ପକାଇବାରେ ଯଦି ଅସୁବିଧା ହେଉଥାଏ ତେବେ ରହିଥିବା ବିଭିନ୍ନ ଦଳଭିତ୍ତିକ ଅନୁଷ୍ଠାନ/ଦଳ ଯଥା GKS ଓ MC/JC ଇତ୍ୟାଦିର ନାମ କୁହନ୍ତୁ?)

7. Have you ever engaged with these groups? If no, why? And if yes, what happened? Prompt: Do you feel they are useful in improving access to food rations and healthcare?
ଏହି ଦଳ ଗୁଡିକ ସାଂଗରେ ଆପଣ ସମ୍ପୃକ୍ତ ହୋଇଛନ୍ତି କି? ଯଦି ନୁହେ କାହିଁକି?ଯଦି ହଁ କଣ ହୋଇଥିଲା?
(ସୂଚାଇ କୁହନ୍ତୁ: ଆପଣ ଭାବୁଛନ୍ତିକି ଖାଦ୍ୟ ରାସନ୍ ଏବଂ ସ୍ବାସ୍ଥ୍ୟ ର ଯତ୍ନ ପାଇଁ ଏଗୁଡିକ ଉପାଦେୟ?)
