# Supplementary material for: The challenges of institutionalizing community-level social accountability mechanisms for health and nutrition: a qualitative study in Odisha, India
Source: BMC Health Serv Res. 2018 Oct 19;18:788. doi: 10.1186/s12913-018-3600-1 (PMC6194642; doi:10.1186/s12913-018-3600-1)
Supplement: Supplementary file 2 — Focus Group Discussion schedule – SHG members. (PDF 614 kb) [file 12913_2018_3600_MOESM2_ESM.pdf]

DRAFT - FGD with SHG Members

Interview code: [ ]

Audio File code: [ ]

Date:

District: Block:

Village: Location of interview:

Interviewer Name: Note Taker Names:

CONSENT OF RESPONDENTS

ଉତ୍ତରଦାତାଙ୍କ ସମ୍ମତି ପ୍ରଦାନ

Thank you for this opportunity to speak with you. Together with the Institute for Development Studies (IDS), we are conducting a survey that will provide us with necessary information to carry out research that is designed to help promote the welfare of people in Odisha; particularly, to improve food consumption and nutrition of the people, and to enhance community accountability and income generation.

ଆପଣଙ୍କ ସହିତ ଆଲୋଚନା କରିବା ପାଇଁ ସୁଯୋଗ ମିଳିଥିବାରୁ ଧନ୍ୟବାଦ ଜଣାଉଛି I ଆମେ institute of development studies (IDS) ସହିତ ଏକ ସର୍ଭେ କାର୍ଯ୍ୟ କରୁଛୁ ଏଥିରୁ ଯେଉଁ ସୁଚନା ସବୁ ମିଳିବ ତାହାକୁ ଆଧାର କରି ଆମ୍ଭେ ଏକ ଅନୁଧ୍ୟାନ ବା research କରିବୁ ଯେଉଁଥିରେ କି ଓଡିଶା ବାସିଙ୍କ ମଗଂଳ ବିଶେଷ କରି ସମାନଙ୍କର ଖାଦ୍ୟା ଖାଇବା ଏବଂ ଲୋକମାନଙ୍କର ପୌଷ୍ଟିକ ସ୍ଥିତି ତଥା ଆୟ ପନ୍ଥା ସୃଷ୍ଟି ଏବଂ ଗୋଷ୍ଟି ଉତ୍ତର ଦାଇତ୍ବ ସ୍ଥାନ ପାଇବ I

We are inviting you to be a participant in this study. We value your opinion and there are no wrong answers to the questions we will be asking in the interview. We will use approximately 45 minutes of your time to collect all the information. There will be no cost to you other than your time. There will be no risk as a result of your participating in the study. Your participation in this research is completely voluntary. You are free to withdraw your consent and discontinue participation in this study at any time.

This study is conducted anonymously. You will only be identified through code numbers. Your identity will not be stored with other information we collect about you. Any information we obtain from you during the research will be kept strictly confidential. This interview will be audio recorded and its content will not be shared or used outside the scope of this research.

ଏହି ଅନୁଧ୍ୟାନରେ ଏକ ଅଂଶ ଗ୍ରହଣ କରି ହେବା ପାଇଁ ଆମେ ଆପଣଙ୍କୁ ଅନୁରୋଧ କରୁଛୁ ଆମେ ପଚାରିବା ଯେ କୌଣସି ପ୍ରଶ୍ନ ପାଇଁ ଆପଣଙ୍କ ମତାମତ କୁ ଆମେ ଗୁରତ୍ବର ସହ ଗ୍ରହଣ କରିବୁ ଆଉ ଏକ କଥା କେଉଛି ଏଥିରେ କୌଣସି ଉତ୍ତର ଭୁଲ୍ ଠିକ୍ ର ବିବେଚନା ମଧ୍ୟ କରଜିବ ନାହିଁ I କଥାଟି ହେଲା ଆପଣ କେବଳ ଏଥିପାଇଁ ସମୟ ଦେବା ବ୍ୟତିତ ଆପଣଙ୍କୁ ଆଉ କିଛି ଦବାର ଆବସ୍ୟାକତା ନାହିଁ I ସୁଚନା ସଂଗ୍ରହ ନିମନ୍ତେ ଆମ୍ଭେ ଆପଣଙ୍କର ୪୫ ମିନିଟ୍ ସମୟ ନେବୁ ଏହି ଅନୁଧ୍ୟାନର ଆପଣଙ୍କ ଅଂଶ ଗ୍ରହଣ ସମ୍ପୁର୍ଣ ଇଛାଧିନ I ଏହି ଅନୁଧ୍ୟାନରେ ଅଂଶ ଗ୍ରହଣ କରିବାର ସମ୍ମତି କୁ ଆପଣ ଯେ କୌଣସି ସମୟରେ ଫେରାଇ ଦେଇ ଅନୁଧ୍ୟାନ ଅଂଶ ଗ୍ରହଣ କରିବୁ ମନା କରିଦେଇ ପାରନ୍ତି I ଅନୁଧ୍ୟାନ ଟିରେ ସମ୍ପୁର୍ଣ ଗୋପନୀୟତା ଭାଷା କରା ଯାଇଛି ଏଥିରେ ଆପଣଙ୍କ ପରିଚୟ ଏକ କୋଡ୍ ନମ୍ବର୍ ହିଁ ରହିବ ଅନ୍ୟ କୌଣସି ସୁଚନା ସହିତ ଆପଣଙ୍କ ପରିଚୟ କୁ ସଲଗ୍ନ କରାଯିବ ନାହିଁ ଅନୁଧ୍ୟାନ ପ୍ରକ୍ରିୟା ରେ ଆପଣଙ୍କ ଠାରୁ ସମ୍ପୁର୍ଣ ଗୋପନ ରଖାଯିବ I ଏହି ସାକ୍ଷାତକାରଟିକୁ ଆମେ ରେକର୍ଡ କରିବାକୁ ଚାହୁଁଚୁ ଏହାକୁ କେବଳ ଆମେ ରିସର୍ଚ୍ ପାଇଁ ବ୍ୟବହାର କରିବୁ , ଅନ୍ୟ କେଉଁଠି ପ୍ରକାଶ କରାଯିବ ନାହିଁ.

Your participation will be highly appreciated. The answers you give will help provide better information to policy-makers, practitioners and program managers so that they can plan for better services that will respond to your needs.

ଅନୁଧ୍ୟାନରେ ଆପଣଙ୍କ ଅଂସ ଗ୍ରହଣ ପ୍ରଶଂଶନିୟ ହୋଇ ରହିବ I ଅନୁଧ୍ୟାନ ମାଧ୍ୟମରେ ଆପଣଙ୍କ ଉତ୍ତର ଓ ସୁଚନା ଗୁଡିକ ନୀତି ନିୟମ ପ୍ରସ୍ତୁତ କରି, କାର୍ଯ୍ୟକାରୀ ପେଶାଦାର, କାର୍ଯ୍ୟକ୍ରମ ପରିଚାଳନା କାରିକୁ ଖୁରାକ ଯୋଗାଇବା, ଫଳତଃ ସେମାନେ ଆପଣଙ୍କ ଉତ୍ତର ମାଧ୍ୟମରେ ଉପସ୍ଥାପନ କରିଥିବା ଆବଶ୍ୟକତାର ପୁରଣ ନିମନ୍ତେ ଓ ସେବା ଯୋଗାଣ ନିମନ୍ତେ ଉତ୍ତମ ଯୋଜନା ପ୍ରସ୍ତୁତ କରିବାରେ ଉତ୍ତମ ଯୋଜନା ଅରିପାରିବେ I

The researcher read to me orally the consent form and explained to me its meaning. I agree to take part in this research. I understand that I am free to discontinue participation at any time if I so choose, and that the investigator will gladly answer any question that arise during the course of the research.

ଅନୁଧାନକାରି ସମ୍ମତି ପତ୍ର କୁ ମୋ ସାମ୍ନାରେ ସମ୍ପୁର୍ଣ ଭାବେ ପଢ଼ି ସଣାଇ ଛନ୍ତି ଏବଂ ଏହାର ଅର୍ଥ ମତେ ବୁଝାଇ ଛନ୍ତି ଏହି ଅନୁଧାନ ରେ ଭାଗ ନେବା ପାଇଁ ମୁ ରାଜି । ମୁ ଭଲଭାବେ ଜନିଛି ଯେ ଯେକୌଣସି ସମୟରେ ମୁ ଅନୁଧାନର ନିଜକୁ ହେରାଇ ଆଣିପାରିବ ଏବଂ ଅନୁଧାନକାରି ଇଣ୍ଟରଭ୍ୟୁ ମଧରେ ଉପୁଜିଥିବା ପ୍ରଶ୍ନ ଗୁଡିକ ର ଉତ୍ତର ଖୁସିରେ ଦେବେ ।

Contact Persons:

ଯୋଗାଯୋଗ ଠିକଣା

Satyanarayan Mohanty, DCOR Consulting

Address: DCOR Consulting Pvt. Ltd., 131 (P), Punjabi Chhak, Satyanagar, Odisha, India, Pin – 751007

Tel: +91-9437698965, E-mail: satya.dcor@gmail.com

Dr. Nicholas Nisbett, IDS

Address: Institute of Development Studies, University of Sussex, Brighton BN1 9RE

Tel: +44 (0)1273 606261; E-mail: n.nisbett@ids.ac.uk

Please tick mark on the right box depending on the respondent's consent

ଉତ୍ତର ଦାତା/ଦାତ୍ରୀ ସମ୍ମତିକୁ ଭିତି କରି ନିରଧିଷ୍ଟ କୋଠରୀରେ ଠିକ୍ ଚିହ୍ନ ଦିଅନ୍ତୁ

Consent given: ସମ୍ମତି ପ୍ରଦାନ

Yes	No

Signature of the Enumerator: ______________________ **Date: DD/_______/_______/_______/**

A. Preliminary Information: ମୌଳିକ ସୁଚନା

A. Preliminary Information : ପ୍ରାରମ୍ଭିକ ସୁଚନା

1. N. of people attending FGD:କତେେ ସଂଖ୍ୟାର ଲେୋକ ଉଦିଷ୍ଟ ଦଳଗତ ଆଲେୋଚନାରେ ଭାଗନେଇଛନ୍ତି ?

	Name of SHG	Years/Months of SHG Existence	Age of Member (in completed years)	Caste/ Ethnicity	Religion	Education
Member 1						
Member 2						
Member 3						
Member 4						
Member 5						
Member 6						
Member 7						
Member 8						

B. SHG Role: SHG ର ଭୁମିକା

1. Could you talk about the SHG formation?

ଆପଣ SHG ଗଠନ ବାବଦରେ କହିପାରିବେ କି ?

ସୁଚାଇ କୁହନ୍ତୁ :ଏହା କେବେ ଓ କିଭଳି ଭାବେ ସଂଗଠନ ହୋଇଥିଲା , ଏବଂ କାହା ଦ୍ବାରା ?

Why did you join the SHG?

ଏଥିରେ କାଂହିକି ଯୋଗ ଦେଲେ ?

2. Could you describe some of the activity the SHG does?

Prompts: What type of activity?

Who is involved and who is it targeted to/benefits from it? (Note: Please inquiry about background of people engaged, i.e. a particular caste or group)

What is the result/impact?

SHG ର କେତେକ କାର୍ଯ୍ୟ ବିଷୟରେ କହିବେ କି?

ସୁଚାଇ କୁହନ୍ତୁ କେଉଁ ପ୍ରକାରର କାର୍ଯ୍ୟକ୍ରମ ? ଏଥିରେ କେଉଁମାନେ ସାମିଲ୍ ଥାନ୍ତି ଏବଂ କେଉଁମାନେ ଏଥହିରୁ ଉପକୃତ ହେବାର ଲକ୍ଷ ରଖାଯାଇଥାଏ ? କେଉଁମାନେ ବାଦ ପଡିଥାନ୍ତି ?

ଦ୍ରଷ୍ଟବ୍ୟ :କେଉଁ ସ୍ତରର ମହିଳାମାନେ ଏଥିରେ ସାମିଲ୍ ଥାନ୍ତି , ପଚାରି ବୁଝନ୍ତୁ ଯାହାକି ଏକ ସ୍ବତନ୍ତ୍ର ଜାତି ବା ବର୍ଗର ମହିଳା ହୋଇପାରନ୍ତି ଫଳା ଫଳ /ପ୍ରଭାବ କଣ ଥିଲା ?

. Why do you think these activities are important to you?

ଏହି କାର୍ଯ୍ୟକ୍ରମ ଗୁଡିକ ଆପଣଙ୍କ ପାଇଁ ଗୁରୁତ୍ବ ପୁର୍ଣ୍ଣ ବୋଲି ଆପଣ କାହିଁକି ଭାବୁଛନ୍ତି ?

C. Participation and decision-making:ଅଂଶ ଗ୍ରହଣ ଓ ନିଷ୍ପତି ଗ୍ରହଣ

1. Could you talk about the last meeting the SHG had?

Prompts: Who convened the meeting? How often are they held? Who attends the meetings?

SHG ର ଗତ ମିଟିଂଗ୍ କେବେ ହୋଇଥିଲା ଆପଣ କହିପାରିବ କି?

ସୁଚାଇ କୁହନ୍ତୁ : ମିଟିଂଗ୍ ରେ କିଏ ଅଧକ୍ଷତା କରିଥିଲେ କେତେ ଦିନ ବ୍ୟବଧାନରେ ଏହି ମିଟିଂଗ୍ ଆୟୋଜିତ ହୁଏ ? କିଏ ମିଟିଂଗ୍ ରେ ଯୋଗ ଦିଅନ୍ତି? କାହିଁକି କିଛି ସଦସ୍ୟା ଅନ୍ୟମାନଙ୍କ ଅପେକ୍ଷା ଅଧିକ ଥର ମିଟିଂଗ୍ ରେ ଯୋଗ ଦେଇଥାନ୍ତି?

2(If was not previously answered: What did you discuss during the last meeting? And what was the outcome of the discussion?)

(ଯଦି ପୁର୍ବ ପ୍ରଶ୍ନର ଉତ୍ତର ନ ଦିଅନ୍ତି) ଗତ ମିଟିଂଗ୍ ରେ ଆପଣ କଣ ସବୁ ଆଲୋଚନା କରିଥିଲେ ଏବଂ ଏହି ଆଲୋଚନା ର ଫଳା ଫଳ କଣ ଥିଲା ?

. During SHG meetings, do you feel comfortable in speaking up or taking part in the discussions?

> If not, why is it so?

Prompt: Who chairs the meeting?

How many people speak or actively participate to discussions? Why do you think some people participate more often than others?

SHG ମିଟିଂଗ୍ ରେ ଆପଣଙ୍କୁ ଭାଗ ନେବାକୁ ଓ କହିବାକୁ ଭଲ ଲଗେକି? ଯଦି ନୁହେଁ ଏପରି କାହିଁ କି ହୁଏ

(ସୁଚାଇ କୁହନ୍ତୁ : ମିଟିଂଗ୍ ରେ କିଏ ଅଧକ୍ଷତା କରନ୍ତି? କେତେ ଲୋକ ସକ୍ରିୟ ଭାବେ କହନ୍ତି ଏବଂ ମିଟିଂଗ୍ ରେ ଅଂଶ ଗ୍ରହଣ କରନ୍ତି କେତେକ ଲୋକ ଅନ୍ୟ ମାନଙ୍କ ତୁଳନାରେ ଅଂସ ଗ୍ରହଣ କରିଥାନ୍ତି ବୋଲି ଆପଣ କାହିଁକି ଭାବଥାନ୍ତି ?

. Have you ever raised an issue or complaint with the SHG?

If yes: what was that about and if not, why you never complained? (Nothing to complain about, afraid to raise issues, no point in raising issues etc)

ଆପଣ କେବେ SHG ର କୌଣସି ସମସ୍ୟା ବାବଦରେ ସ୍ବର ଉତ୍ତୋଳନ କରିଛନ୍ତି କି?

ଯଦି ହଁ ତେବେ ଏହା କେଉଁ ବାବଦରେ ଥିଲା? ଯଦି ନା ତେବେ ଏପରି କାହିଁକି ହେଲା ଏବଂ ଆପଣ କାହିଁକି ଅଭିଯୋଗ କଲେ ନାହିଁ?

(କୌଣସି ବିଷୟରେ ଅଭିଯୋଗ କରିବାରେ କିଛି ନାହିଁ, ସମସ୍ୟା କହିବାରେ କିଛି ଭୟ କରିବାର ନାହିଁ, ସମସ୍ୟା କହିବାକୁ ସେମିତି କିଛି ବିଷୟ ନାହିଁ)

D. Community Mobilization on health and nutrition

ସ୍ବାସ୍ଥ୍ୟ ଏବଂ ପୃଷ୍ଟି ରେ ଗୋଷ୍ଠି ସଂଚାଳନ

1. What are the main challenges you face *as women*?

ଜଣେ ମହିଳା ଭାବେ ଆପଣ କେଉଁ ସବୁ ମୁଖ୍ୟ ପ୍ରତିବନ୍ଧକକୁ ସାମ୍ନା କରିଥାନ୍ତି?

2. Do you feel that women from other communities face the same issues? What's the difference?

ବିଭିନ୍ନ ଜାତି ଏବଂ ଜନ ଜାତିର ମହିଳା ମାନେ ସେହି ସମାନ ସମସ୍ୟାକୁ/ପ୍ରତିବନ୍ଧକ କୁ ସାମନା କରିଥାନ୍ତି ବୋଲି ଆପଣ ଭାବୁଛନ୍ତିକି? ତେବେ ଏଥିରେ କି ଫରକ ରହିଥାଏ?

3. How does the work of the SHG promote women's social status and rights? Please make examples!

SHGର କାର୍ଯ୍ୟକ୍ରମ କେଉଁଭଳି ଭାବେ ମହିଳାମାନଂକର ସମାଜିକସ୍ତିତି ଏବଂ ଅଧିକାରରେ ଉନ୍ନତି ଆଣିଥାଏ?

Prompts: For instance, awareness raising campaigns, income-generation activities, support with accessing food/health services, knowledge of entitlements/service delivery etc.

(ସୁଚାଇ କୁହନ୍ତୁ: ଉଦାହରଣ ସ୍ବରୁପ ସଚେତନତା କାର୍ଯ୍ୟକ୍ରମ, ଆୟପନ୍ଥା କାର୍ଯ୍ୟକ୍ରମ, ଖାଦ୍ୟ/ସ୍ବାସ୍ଥ୍ୟସେବା ପାଇବାର ଅଧିକାର/ସେବାଯୋଗାଣ ବ୍ୟବସ୍ଥା ବାବଦରେ ଜ୍ଞାନ ଦେବ ଇତ୍ୟାଦି)

4. What do you see as the most pressing health and nutrition issues among women in your village? Does the SHG work on these issue? If not, why? And if yes, what is its role?

ଆପନଂକ ଗ୍ରାମରେ ମହିଳା ମାନଂକର କେଉଁ ସ୍ବାସ୍ଥ୍ୟଗତ ଓ ପୃଷ୍ଟିଗତ ସମସ୍ୟାଟି ଅତିସୟ ବୋଲି ଆପଣ ଅନୁଭବ କରିପାରୁଛନ୍ତି? SHG ଏ ଦିଗରେ କିଛି କାର୍ଯ୍ୟ କରୁଛି କି? ଯଦି ନୁହେଁ କାହିଁକି? ଏବଂ ଯଦି ହଁ ଏହାର ଭୁମିକା କଣ?

(Only if difficulty in answering, probe examples like: lack of ambulance, lack of medical staff, unawareness about health issues, not enough to eat etc. Please inquiry about perceive root causes of these issues)

(ଯଦି ଉତ୍ତରଦେବା କଷ୍ଟକର ହୁଏ ଉଦାହରଣ ମାଧମରେ ସୁଚାଇ କୁହନ୍ତୁ: ଏହା ଆମ୍ବୁଲାନ୍ସ୍ ଜନିତ ଅଭାବରୁ, ମେଡିକାଲ୍ ରେ କର୍ମଚାରୀ ନଥିବାରୁ ସ୍ବାସ୍ଥ୍ୟ ସମସ୍ୟା ବବଦରେ ସଚେତନତା ଅଭାବରୁ, ପରିମିତ ଖାଇବାର ସମସ୍ୟାର. ଦୟାକରି ଏଗୁଡିକୁ ପଚାରିବୁଝନ୍ତୁ.? ଏହି ସମସ୍ୟା ଗୁଡିକର ମୁଳକାରଣ କଣ ହୋଇଥାଇପାରେ)

5. How does the SHG engage with pregnant and lactating women and young children in your village?
Prompts: What are some of the things you tell women when you speak to them ?
Who exactly do you engage with? (Note: Please inquiry about background of people engaged, i.e. a particular caste or group)
ଗାଁ ରେ ଗର୍ଭବତୀ, ପ୍ରସୂତି, ଏବଂ ବଢ଼ାନ୍ତ ଶିଶୁଙ୍କ ମା ମାନଙ୍କ ସହିତ SHG ର କିପରିଭାବେ ସାମିଲ୍ ହୋଇ କାମ କରିଥାଏ? ସୁଚାଇ କୁହନ୍ତୁ:ମହିଳା ମାନଙ୍କ ସହିତ କିଛି କାର୍ଯ୍ୟକ୍ରମ ବେଳେ ଆପଣ କେଉଁ ମୁଖ୍ୟ କାର୍ଯ୍ୟ ସବୁ ଜଣାଇଥାଆନ୍ତି?
ପ୍ରକୃତରେ କେଉଁମାନଂକୁ ନେଇ ଆପଣ କାର୍ଯ୍ୟ କରିଥାନ୍ତି?(ଦ୍ରଷ୍ଟବ୍ୟ: ପଚାରି ବୁଝନ୍ତୁ ପ୍ରକୃତରେ କେଉଁ ଶ୍ରେଣୀର ମହିଳାକୁ ସାମିଲ୍ କରାଯାଇଥାଏ? କିଛି ସ୍ବତନ୍ତ୍ର ଜାତି/ଜନଜାତିର ମହିଳା)

6. How do you think the SHG could do more? What else is needed?
SHG ଆଉ ଅଧିକ କିଛି କରିପାବବୋଲି ଆପଣ ଭାବୁଛନ୍ତି କି? ଏଥିପାଇଁ କ'ଣ ସବୁ ଆବଶ୍ୟକ?
