# Supplementary material for: The challenges of institutionalizing community-level social accountability mechanisms for health and nutrition: a qualitative study in Odisha, India
Source: BMC Health Serv Res. 2018 Oct 19;18:788. doi: 10.1186/s12913-018-3600-1 (PMC6194642; doi:10.1186/s12913-018-3600-1)
Supplement: Supplementary file 3 — In-Depth Interview Schedule - ASHA or AWW. (PDF 596 kb) [file 12913_2018_3600_MOESM3_ESM.pdf]

DRAFT - IDI with ASHA or AWW

Interview code: ☐

Audio File code: ☐

Date:

District: Block:

Village: Location of interview:

Interviewer Name: Note Taker Names:

CONSENT OF RESPONDENT

<u>ଉତ୍ତରଦାତାଙ୍କ ସମ୍ମତି ପ୍ରଦାନ</u>

Thank you for this opportunity to speak with you. Together with the Institute for Development Studies (IDS), we are conducting a survey that will provide us with necessary information to carry out research that is designed to help promote the welfare of people in Odisha;particularly,to improve food consumption and nutrition of the people, and to enhance community accountability and income generation.

ଆପଣଙ୍କ ସହିତ ଆଲୋଚନା କରିବା ପାଇଁ ସୁଯୋଗ ମିଳିଥିବାରୁ ଧନ୍ୟବାଦ ଜଣାଉଛି । ଆମେ institute of development studies (IDS) ସହିତ ଏକ ସର୍ଭେ କାର୍ଯ୍ୟ କରୁଛୁ ଏଥିରୁ ଯେଉଁ ସୁଚନା ସବୁ ମିଳିବ ତାହାକୁ ଆଧାର କରି ଆମ୍ଭେ ଏକ ଅନୁଧ୍ୟାନ ବା research କରିବୁ ଯେଉଁଥିରେ କି ଓଡିଶା ବାସିଙ୍କ ମଗଂଳ ବିଶେଷ କରି ସମାନଙ୍କଂର ଖାଦ୍ୟା ଖାଇବା ଏବଂ ଲୋକମାନଙ୍କଂର ପୌଷ୍ଟିକ ସ୍ଥିତି ତଥା ଆୟ ପନ୍ଥା ସୃଷ୍ଟି ଏବଂ ଗୋଷ୍ଠି ଉତ୍ତର ଦାଇତ୍ବ ସ୍ଥାନ ପାଇବା

We are inviting you to be a participant in this study. We value your opinion and there are no wrong answers to the questions we will be asking in the interview. We will use approximately 45 minutes of your time to collect all the information. There will be no cost to you other than your time.There will be no risk as a result of your participating in the study. Your participation in this research is completely voluntary. You are free to withdraw your consent and discontinue participation in this study at any time.

This study is conducted anonymously. You will only be identified through code numbers. Your identity will not be stored with other information we collect about you.Any information we obtain from you during the research will be kept strictly confidential. This interview will be audio recorded and its content will not be shared or used outside the scope of this research.

ଏହି ଅନୁଧ୍ୟାନରେ ଏକ ଅଂଶ ଗ୍ରହଣ କରି ହେବା ପାଇଁ ଆମେ ଆପଣଙ୍କୁ ଅନୁରୋଧ କରୁଛୁ ଆମେ ପଚାରିବା ଯେ କୌଣସି ପ୍ରଶ୍ନ ପାଇଁ ଆପଣଙ୍କ ମତାମତ କୁ ଆମେ ଗୁରତ୍ବର ସହ ଗ୍ରହଣ କରିବୁ ଆଉ ଏକ କଥା କେଉଛି ଏଥିରେ କୌଣସି ଉତ୍ତର ଭୁଲ୍ ଠିକ୍ ର ବିବେଚନା ମଧ କରଜିବ ନାହିଁ । କଥାଟି ହେଲା ଆପଣ କେବଳ ଏଥିପାଇଁ ସମୟ ଦେବା ବ୍ୟତିତ ଆପଣଙ୍କୁ ଆଉ କିଛି ଦବାର ଆବସ୍ୟାକତା ନାହିଁ । ସୁଚନା ସଂଗ୍ରହ ନିମନ୍ତେ ଆମ୍ଭେ ଆପଣଙ୍କର ୪୫ ମିନିଟ୍ ସମୟ ନେବୁ ଏହି ଅନୁଧ୍ୟାନର ଆପଣଙ୍କ ଅଂଶ ଗ୍ରହଣ ସମ୍ପୁର୍ଣ ଇଛାଧିନ । ଏହି ଅନୁଧ୍ୟାନରେ ଅଂଶ ଗ୍ରହଣ କରିବାର ସମ୍ମତି କୁ ଆପଣ ଯେ କୌଣସି ସମୟରେ ଫେରାଇ ଦେଇ ଅନୁଧ୍ୟାନ ଅଂଶ ଗ୍ରହଣ କରିବୁ ମନା କରିଦେଇ ପାରନ୍ତି । ଅନୁଧ୍ୟାନ ଟିରେ ସମ୍ପୁର୍ଣ ଗୋପନୀୟତା ଭାଷା କରା ଯାଇଛି ଏଥିରେ ଆପଣଙ୍କ ପରିଚୟ ଏକ କୋଡ୍ ନମ୍ବର୍ ହିଁ ରହିବ ଅନ୍ୟ କୌଣସି ସୁଚନା ସହିତ ଆପଣଙ୍କ ପରିଚୟ କୁ ସଲଗ୍ନ କରାଯିବ ନାହିଁ ଅନୁଧ୍ୟାନ ପ୍ରକ୍ରିୟା ରେ ଆପଣଙ୍କ ଠାରୁ ସମ୍ପୁର୍ଣ ଗୋପନ ରଖାଯିବ । ଏହି ସାକ୍ଷାତକାରଟିକୁ ଆମେ ରେକର୍ଡ କରିବାକୁ ଚାହୁଁଚୁ ଏହାକୁ କେବଳ ଆମେ ରିସର୍ଚ୍ ପାଇଁ ବ୍ୟବହାର କରିବୁ , ଅନ୍ୟ କେଉଁଠି ପ୍ରକାଶ କରାଯିବ ନାହିଁ.

Your participation will be highly appreciated. The answers you give will help provide better information to policy-makers, practitioners and program managers so that they can plan for better services that will respond to your needs.

ଅନୁଧ୍ୟାନରେ ଆପଣଙ୍କ ଅଂସ ଗ୍ରହଣ ପ୍ରଶଂଶନିୟ ହୋଇ ରହିବ । ଅନୁଧ୍ୟାନ ମାଧ୍ୟମରେ ଆପଣଙ୍କ ଉତ୍ତର ଓ ସୁଚନା ଗୁଡିକ ନୀତି ନିୟମ ପ୍ରସ୍ତୁତ କରି, କାର୍ଯ୍ୟକାରୀ ପେଶାଦାର, କାର୍ଯ୍ୟକ୍ରମ ପରିଚାଳନା କାରିକୁ ଖୁରାକ ଯୋଗାଇବା, ଫଳତଃ ସେମାନେ ଆପଣଙ୍କ ଉତ୍ତର ମାଧ୍ୟମରେ ଉପସ୍ଥାପନ କରିଥିବା ଆବଶ୍ୟକତାର ପୁରଣ ନିମନ୍ତେ ଓ ସେବା ଯୋଗାଣ ନିମନ୍ତେ ଉତ୍ତମ ଯୋଜନା ପ୍ରସ୍ତୁତ କରିବାରେ ଉତ୍ତମ ଯୋଜନା ଅରିପାରିବେ ।

The researcher read to me orally the consent form and explained to me its meaning.I agree to take part in this research. I understand that I am free to discontinue participation at any time if I so choose, and that the investigator will gladly answer any question that arise during the course of the research.

ଅନୁଧ୍ୟାନକାରି ସମ୍ମତି ପତ୍ର କୁ ମୋ ସାମ୍ନାରେ ସମ୍ପୂର୍ଣ ଭାବେ ପଢ଼ି ଜଣାଇଛନ୍ତି ଏବଂ ଏହାର ଅର୍ଥ ମତେ ବୁଝାଇଛନ୍ତି ଏହି ଅନୁଧ୍ୟାନ ରେ ଭାଗ ନେବା ପାଇଁ ମୁଁ ରାଜି । ମୁଁ ଭଲଭାବେ ଜାଣିଛି ଯେ ଯେକୌଣସି ସମୟରେ ମୁଁ ଅନୁଧ୍ୟାନର ନିଜକୁ ଫେରାଇ ଆଣିପାରିବ ଏବଂ ଅନୁଧ୍ୟାନକାରି ଇଣ୍ଟରଭ୍ୟୁ ମଧ୍ୟରେ ଉପୁଜିଥିବା ପ୍ରଶ୍ନଗୁଡିକ ର ଉତ୍ତର ଖୁସିରେ ଦେବେ ।

Contact Persons:

ଯୋଗାଯୋଗ ଠିକଣା

Satyanarayan Mohanty, DCOR Consulting

Address: DCOR Consulting Pvt. Ltd., 131 (P), Punjabi Chhak, Satyanagar, Odisha, India, Pin – 751007

Tel: +91-9437698965, E-mail: satya.dcor@gmail.com

Dr.Nicholas Nisbett, IDS

Address: Institute of Development Studies, University of Sussex, Brighton BN1 9RE

Tel: +44 (0)1273 606261; E-mail: n.nisbett@ids.ac.uk

Please tick mark on the right box depending on the respondent's consent

ଉତ୍ତର ଦାତା/ଦାତ୍ରୀ ସମ୍ମତିକୁ ଭିତି କରି ନିରଧିଷ୍ଟ କୋଠରୀରେ ଠିକ୍ ଚିହ୍ନ ଦିଅନ୍ତୁ

Consent given: ସମ୍ମତି ପ୍ରଦାନ	Yes	No

Signature of the Enumerator: ______________________Date: DD/_______/_______/_______/

A. Preliminary Information: ମୌଳିକ ସୁଚନା

1. Age:ବୟସ :

2. Gender:ଲିଂଗ :

3. Years/months in service in this position:
ଉକ୍ତ ପଦବୀରେ କେତେ ମାସ/ବର୍ଷ ହେଲା ସେବା ଯୋଗାଉଛନ୍ତି |

4 Caste/Ethnicity: ଜାତି/ପାରମ୍ପରିକ ବର୍ଗ

5. Religion: ଧର୍ମ

6. Education: ଶିକ୍ଷା

7. Committee(s) of the interviewee: ଉତ୍ତରଦାତା କେଉଁ କମିଟିଗୁଡିକରେ ଅଛନ୍ତି |

A. Name of Committee: __________________________ Position held: _____________________Years/months in the position: __________

କମିଟିର ନାମ______________________________________ କେଉଁ ପଦବୀରେ ଅଛନ୍ତି______________________
ଉକ୍ତପଦବୀରେ କେତେ ମାସ/ବର୍ଷ ହେବ ରହିଛନ୍ତି _______________

B. Name of Committee: __________________________ Position held: _____________________
Years/months in the position: ___________________

କମିଟିର ନାମ______________________________________କେଉଁ ପଦବୀରେ ଅଛନ୍ତି______________________
ଉକ୍ତପଦବୀରେ କେତେ ମାସ/ବର୍ଷ ହେବ ରହିଛନ୍ତି _______________

C. Name of Committee: __________________________ Position held: _____________________
Years/months in the position: __________

କମିଟିର ନାମ______________________________________କେଉଁ ପଦବୀରେ ଅଛନ୍ତି______________________
ଉକ୍ତପଦବୀରେ କେତେ ମାସ/ବର୍ଷ ହେବ ରହିଛନ୍ତି _______________

D. Other affiliations: ଅନ୍ୟାନ୍ୟ ସମ୍ପୃକ୍ତି

ଦ୍ରଷ୍ଟବ୍ୟ: ଏଥିମଧ୍ୟରେ ପଂଚାୟତିରାଜ୍ ଅନୁଷ୍ଠାନ, ସରକାରୀ ସଂସ୍ଥା ଗୋଷ୍ଠି ଭିତିକ ଅନୁଷ୍ଠାନ ଏବଂ ସେଛାସେବିଅନୁଷ୍ଠାନ ଅନ୍ତର୍ଭୁକ୍ତ

____

Note: Include affiliations with PRI, Government agencies, CBOs and NGOs.

B. MC ମାତୃ କମିଟି ଏବଂ ଯାଂଚ୍ କମିଟି

B.1) Role and effectiveness କମିଟିର ଭୁମିକା ଏବଂ ଫଳପ୍ରଦ

1. Could you please describe some of the activity the MC and JC do in your village?

Note: Please ask about what MC/JC *actually* does in the village, i.e. not guidelines-type

answer, but tasks that are actually done.
Please try to get specific by prompting 'how?' after the answer (if needed).
ମାତୃ କମିଟି ଏବଂ ଯାଂଚ୍ କମିଟି କରୁଥିବା କେତେକ କାର୍ଯ୍ୟ ବିଷୟରେ ବୁଝାଇ କହିବେ କି ?
ଦ୍ରଷ୍ଟବ୍ୟ: ଏହି କାର୍ଯ୍ୟରେ କେଉଁମାନେ ସାମିଲ୍ ହୋଇଥାନ୍ତି? ଏଥିରୁ କେଉଁମାନେ ଉପକୃତ ହୁଅନ୍ତି?

2. What are the challenges in executing some of these activities?
Note: Please help respondent elaborate on the answer, for instance by asking for examples.
ଏହି କାର୍ଯ୍ୟଗୁଡିକରୁ କରିବାରେ ଆପଣ କେଉଁସବୁ ପ୍ରତିବନ୍ଧକର ସାମ୍ନା କରିବାକୁପଡେ ?

3. What is your role in the Committees?
(Note: what do you *actually* do in the Committee)
କମିଟିରେ ଆପଣଙ୍କର ଭୂମିକା କଣ ?
ଦ୍ରଷ୍ଟବ୍ୟ:ଆପଣ କମିଟିରେ କଣସବୁ କରିଥାନ୍ତି ?

4. What is the impact of the Committee on your work as an ASHA/AWW? Do you feel the committee is helping your work or making it more difficult? Why is that?
ଜଣେ ASHA / AWW ଭାବେ କାର୍ଯ୍ୟକରିବାରେ କମିଟିର କଣ ପ୍ରଭାବ ରହିଚି ?ଆପଣ ଭାବୁଛନ୍ତି କି ଆପଣଙ୍କ କାର୍ଯ୍ୟକୁ କମିଟି ସାହାଯ୍ୟ କରୁଛି ବା ଜଟିଳ କରୁଛି କି? ଏପରି କାହିଁକି ହେଉଛି?

B.2) Participation and decision-making/ଅଂଶ ଗ୍ରହଣ ଏବଂ ନିଷ୍ପତି ଗ୍ରହଣ

1. Who are the other members of the Committee? How were they appointed in your village?
କେଉଁମାନେ କମିଟିର ଅନ୍ୟ ସଦସ୍ୟ/ସଦସ୍ୟା ଅଛନ୍ତି ? କିପରି ଭାବେ ସେମାନେ ନିୟୋଜିତ ହେଉଛନ୍ତି ?

2. Could you talk about the last meeting you attended?
ଆପଣ ଅଂଶ ଗ୍ରହଣ କରିଥିବା ଗତ ବୈଠକ ବିସୟରେ କହି ପାରିବେ କି ?
Prompts: Who convened the meeting, how often are they usually held and how often do you usually attend
ସୁଚେଇ କୁହନ୍ତୁ :କିଏ ମିଟିଙ୍ଗିରେ ଅଧକ୍ଷତା କରନ୍ତି, ପ୍ରାୟ କେତେ ଦିନରେ ଥରେ ମିଟିଙ୍ଗିବସିଥାଏ, ଏବଂ ଆପଣ କେତେ ବ୍ୟବଧାନରେ ମିଟିଙ୍ଗି ରେ ଯୋଗଦେଇଥାନ୍ତି ?
(If not answered previously: What did you discuss during the last meeting? And what was the outcome of the discussion?)
ଗତ ମିଟିଙ୍ଗିରେ ଆପଣ କଣ ସବୁ ଅଲୋଚନା କରିଥିଲେ? ଏହି ଆଲୋଚନାର ଫଳାଫଳ କଣ ଥିଲା ?

3. What do you think are the barriers other Committee members face in a) organizing meetings b) attending meetings and c) participating?
(Note: enquiry about non-tangible barriers like caste and gender representation and social status of committee members)
ଅନ୍ୟ କମିଟି ସଦସ୍ୟ/ ସଦସ୍ୟା ମାନେ କେଉଁ ସବୁ ପ୍ରତିବନ୍ଧକର ସମ୍ମୁଖିନ ହେଉଛନ୍ତି ବୋଲି ଆପଣ ଭାବୁଛନ୍ତି ?
ସୁଚାଇ କୁହନ୍ତୁ : ବିଶେଷ କରି ଏହି ସବୁ ପ୍ରତିବନ୍ଧକ ଗୁଡିକ (କ) ମିଟିଙ୍ଗି ଆୟୋଜନ କରିବାରେ (ଖ)ମିଟିଂଗରେ ଯୋଗଦେବାରେ ଏବଂ ଅଂଶ ଗ୍ରହଣ କରିବାରେ ଇତ୍ୟାଦି ?

ଦ୍ରଷ୍ଟବ୍ୟ: ଖାଲି ଆଖିରେ ଦେଖାଯାଉନଥିବା ଯଥା ଜାତି ଏବଂ ଲିଂଗ ଭେଦ ଓ ସମସ୍ୟା ସ୍ଥିତି ସମ୍ବନ୍ଧରେ ପ୍ରତିବନ୍ଧକ ବାବଦରେ ପଚାରି ବୁଝିଲୁ ?

<u>C. GKS</u> ଗାଁ କଲ୍ୟାଣ କମିଟି

<u>C.1) Role and effectiveness:ଭୁମିକା ଏବଂ ଫଳ ପ୍ରଦତା</u>

1. Could you please describe some of the activity the GKS does in your village? Please be specific or provide examples.
Prompts: Who is involved in these activities? Who benefits from them?
ଗାଁ କଲ୍ୟାଣ ସମିତି କରୁଥିବା କାର୍ଯ୍ୟ ବାବଦରେ ଆମକୁ ଦୟାକରି କହିବେ କି ?ସୁଚାଇ କୁହନ୍ତୁ : ଏହି କାର୍ଯ୍ୟ ଗୁଡିକ କରିବାରେ ସାଧାରଣତଃ କେଉଁମାନେ ସାମିଲ୍ ହୋଇଥାନ୍ତି? ଏଥିରୁ କେଉଁମାନେ ଉପକୃତ ହୁଅନ୍ତି ?

2. What is your role in the Committees?
(Note: what do you *actually* do in the Committee in your village)
କମିଟିରେ ଆପଣଙ୍କର ଭୁମିକ କଣ ?ଦ୍ରଷ୍ଟବ୍ୟ:ଆପଣ ପ୍ରକୃତ ପକ୍ଷେ କଣ କରିଥାନ୍ତି

3. What is the impact of the Committee on your work as an ASHA/AWW? (Note: inquiry about whether and how the Committee places extra burden or helps e.g. in service delivery and community outreach) Why you think is it so?
ଜଣେ ASHA / AWW ଭାବେ କାର୍ଯ୍ୟକରିବାରେ କମିଟିର କଣ ପ୍ରଭାବ ରହିଚି ?ଆପଣ ଭାବୁଛନ୍ତି କି ଆପଣଙ୍କ କାର୍ଯ୍ୟକୁ କମିଟି ସାହାଯ୍ୟ କରୁଛି ବା ଜଟିଳ କରୁଛି କି?
(ସେବା ପ୍ରଦାନ ଏବଂ ସେବା ଯୋଗାଇବା ଇତ୍ୟାଦିରେ) ଏପରି ଭାବୁଛନ୍ତି କାହିଁକି?

4. What are some of the challenges in executing some of these activities?Please help respondents elaborate on the answers by asking how/why as a prompt.
ଏଥିରୁ କିଛ କାର୍ଯ୍ୟ ଦେଖାଶୁଣା କରିବା ଓ କରାଇବାରେ କେଉଁସବୁ ପ୍ରତିବନ୍ଧକର ସାମ୍ନା କରିବାକୁ ପଡିଥାଏ?

<u>C.2) Participation and decision-making:ଅଂଶ ଗ୍ରହଣ ଓ ନିଷ୍ପତି ଗ୍ରହଣ</u>

1. Who are the other members of the Committee? How were they appointed?
କମିଟିରେ ଅନ୍ୟ କେଉଁ ସଦସ୍ୟ, ସଦସ୍ୟା ମାନେ ରହୁଛନ୍ତି ? ସେମାନେ କିପରି ନିଯୁକ୍ତି ପାଇଥିଲେ ?

2. Could you talk about the last meeting you attended?
Prompts: Who convened the meeting, how often are they usually held and who attends?
ଆପଣ ଯୋଗଦେଇଥିବା ଗତ ବୈଠକ ବାବଦରେ ସୁଚାଇ କହିବେକି ?
ସୁଚାଇ କୁହନ୍ତୁ : ଏହି ବୈଠକରେ କିଏ ଅଧକ୍ଷତା କରିଥିଲେ, ଏହି ବୈଠକ କେତେଦିନ ବ୍ୟବଧାନରେ ଆୟୋଜିତ ହୋଇଥାଏ ଏବଂ ଏଥିରେ କେଉଁମାନେ ଯୋଗ ଦିଅନ୍ତି?

(If was not answered previously: What did you discuss during the last meeting? And what was the outcome of the discussion?)
ଗତ ବୈଠକରେ ଆପଣ କଣ ସବୁ ଆଲୋଚନା କଲେ ? ଏବଂ ଏହି ଆଲୋଚନାର ଫଳା ଫଳ କଣ ଥିଲା ?

3. What do you think are the barriers other Committee members face in a) organizing meetings b) attending meetings and c) participating?
(Note: enquiry about non-tangible barriers such as caste and gender representation and social status of committee members)
କମିଟିରେ ଥିବା ଅନ୍ୟ ସଦସ୍ୟ ସଦସ୍ୟା ମନେ କେଉଁ ସବୁ ପ୍ରତିବନ୍ଧକର ସମ୍ମୁଖିନ ହେଉଛନ୍ତି ବୋଲି ଆପଣ ଭାବୁଛନ୍ତି ?
ସୁଚାଇ କୁହନ୍ତୁ : ବିଶେଷ କରି ପ୍ରତିବନ୍ଧକ ଗୁଡିକ କ) ବୈଠକ ଆୟୋଜନ କରିବାରେ ଖ) ବୈଠକରେ ଯୋଗଦେବାରେ ଏବଂ ଗ) ଅଂସ ଗ୍ରହଣ କରିବାର ?
ଦ୍ରଷ୍ଟବ୍ୟ: ପଚାରି ବୁଝନ୍ତୁ , ଖାଲି ଆଖିକୁ ଦେଖାଯାଉନଥିବା କେଉଁ ସବୁ ପ୍ରତିବନ୍ଧକ ଯଥା –ଲିଂଗ ଓ ଜାତି ଭେଦ ଭିତିକ ପ୍ରତିନିଧିତ୍ବ ଏବଂ କମିଟି ସଦସ୍ୟ ସଦସ୍ୟାଙ୍କର ସାମାଜିକ ସ୍ଥିତି ଇତ୍ୟାଦି

C.3 Funding : ପାଣ୍ଠିଯୋଗାଣ

1. Has the Committee you are part of received any funding in the last two years?
ଆପଣ ଥିବା କମିଟି ଗତ ଦୁଇ ବର୍ଷ ମଧ୍ୟରେ କଛି ଅନୁଦାନ ପାଣ୍ଠି ପାଇଛି କି ?

2. If so, how was it spent?
ଯଦି ପାଇଥିଲା , ଏହି ପାଣ୍ଠି କିପରି ଖର୍ଚ ହୋଇଛି ?
Prompt: On which heads the funds were spent?
କେଉଁ ବାବଦରେ ପାଣ୍ଠି ଖର୍ଚ୍ଚ ହୋଇଛି ?

3. What are the factors considered when deciding about expenditure?
କେଉଁ ବାବଦରେ ଖର୍ଚ୍ଚ କରାଯିବ ବୋଲି ତାହା ନିଷ୍ପତି କେତବେଳେ ନିଆଯାଏ|
4. How has the expenditure been documented or recorded? (Observation)
ଏହି ଖର୍ଚ ଗୁଡିକରେ ବିବରଣୀ କିପରି ଭାବେ ଟିପି ରଖାଯାଇଛି?(ନିରୀକ୍ଷଣ କରିବା)

D. Coordination :ସଂଯୋଜନ

1. What are the linkages between these committees and health authorities (e.g. ICDS Supervisor and/or Block Programme Manager) How do they keep informed about Committees' activities?
ସ୍ବାସ୍ଥ୍ୟ କର୍ମ କର୍ତ୍ତା ଏବଂ କମିଟି ମଧ୍ୟରେ କେଉଁ ସବୁ ସଂପର୍କ ରହିଛି (ଉଦାହରଣ ସ୍ବରୁପ :ଆଇ.ସି.ଡି.ଏସ ସୁପାରଭାଇଜର୍ ଏବଂ ବ୍ଲକ୍ ପ୍ରୋଗ୍ରାମ୍ ମ୍ୟାନେଜର)କମିଟି ର କାର୍ଯ୍ୟ କଳାପ ବାବଦରେ ତାଙ୍କୁ କିପରି ଭାବରେ ସୁଚନା ଦିଆଯାଇଥାଏ ?

2. Is there an SHG in your village? What is your role in the SHG?
ଆପଣଙ୍କ ଗାଁ ରେ କୌଣସି SHG ଅଛି କି ?ଏହି SHG ରେ ଆପଣଙ୍କ ଭୁମିକା କଣ ?

3. What do you think is the main difference between the SHG and other committees?
ଅନ୍ୟ କମିଟି ଏବଂ SHGମଧ୍ୟରେ ମୁଖ୍ୟତଃ କେଉଁ ସବୁ ତଫାତ ରହିଥାଏ ?

4. What do you think is the role of the SHG in the community?
ସମୁଦାୟରେ SHG ର ଭୁମିକା କଣ ବୋଲି ଆପଣ ଭାବୁଛନ୍ତି ?

E. Community Mobilization on health and nutritionସ୍ୱାସ୍ଥ୍ୟ ଏବଂ ପୁଷ୍ଟି ବାବଦରେ କମିଟି ସଂଚାଳନ

1. What do you think are some of the main issues women, especially mothers, in your village face?
ଆପଣ ଗ୍ରାମରେ ମହିଳା ମାନଙ୍କ ର ବିଶେଷ କରି ମା ମାନଙ୍କର ମୁଖ୍ୟ ସମସ୍ୟାଗୁଡିକ କଣ ବୋଲି ଆପଣ ଭାବୁଛନ୍ତି?

Please help respondent elaborate on the answers by asking how/why or providing examples
(ଦୟାକରି ଉତ୍ତରଦତ୍ତା ଙ୍କ ଉଦାହରଣ ଦେଇ ଏବଂ କିପରି ଓ କାଂହିକି ପଚାରନ୍ତୁ)

2. What do you feel is the root cause for these issues?
ଏହି ସମସ୍ୟା ଗୁଡିକର ମୁଖ୍ୟ କାରଣ ବାବଦରେ ଆପଣ କଣ ଭାବୁଛନ୍ତି ?

3. Could you please explain how these issues impact access to services for people?

ଦୟାକରି କହିବେ କି ? ଲୋକମାନଂକ ପାଖରେ ସେବା ପହଂଚିବା ପାଇଁ ଏହି ସମସ୍ୟାଗୁଡିକ କିପରି ପ୍ରଭାବ ପକାଉଛି.

4. Do you feel these Committees have a role in addressing them? And if so, how?
ଏହି ସମସ୍ୟା ସମାଧାନ ରେ କମିଟି ର ଭୂମିକା ରହିଛି ବୋଲି ଆପଣ ଭାବୁଛନ୍ତି କି ? ଯଦି ଅଛି ତେବେ ଏହା କିପରି ହୋଇପାରେ ?

5. What do you feel is the impact of the Committee in promoting women's health?
Prompts: For instance, how is improved your status within the community? Or how has it improved access to food/health services, knowledge of entitlements/service delivery
ମହିଳା ଙ୍କ ସ୍ୱାସ୍ଥ୍ୟ ଅବସ୍ଥାରେ ଉନ୍ନତି ଆଣିବାରେ କମିଟିର କଣ ପ୍ରଭାବ ପଡିଛି ବୋଲି ଆପଣ ଭାବୁଛନ୍ତି ?
ସୁଚାଇ କୁହନ୍ତୁ ଉଦାହରଣ ସ୍ୱରୁପ କମିଟିରେ ଆପଣଙ୍କ ହିତରେ କିପରି ଉନ୍ନତି ଘଟିଛି ? ଖାଦ୍ୟ/ସ୍ୱାସ୍ଥ୍ୟ ସେବା/ଅଧିକାର ସେବା ଯୋଗାଣ ବାବଦରେ ଜ୍ଞାନ ଆଦି ବାବଦରେ ଅଧିକଉନ୍ନତି ହୋଇଛି ?

6. How does the committee engage with pregnant women and mothers of young children?
What do you tell women to encourage to avail of services?
What are the main challenges you encounter when speaking to women? Please give examples.
କମିଟି ,ଗର୍ଭବତୀ, ପ୍ରଶୁତି ଏବଂ ବଢାନ୍ତା ଶିଶୁ ଙ୍କ ମା ମାନଙ୍କ ପାଇଁ କିପରି କାମ କରୁଛି ?ସୁଚାଇ କୁହନ୍ତୁ: ମହିଳାଙ୍କୁ ନେଇ କାମ କରିବା ବେଳେ ଆପଣ ମୁଖ୍ୟତଃ କଣ ଜଣାଇଥାନ୍ତି?(ଦ୍ରଷ୍ଟବ୍ୟ:ସାମିଲ୍ ହୋଇଥିବା ବ୍ୟକ୍ତି ମାନଙ୍କର ସ୍ଥିତି ବାବଦରେ ପଚାରି ବୁଝନ୍ତୁ ଯାହାକି ଏକ ସ୍ୱତନ୍ତ୍ର ଜାତି ବା ଦଳ/ସମ୍ପ୍ରଦାୟର ଲୋକମାନଙ୍କ) କେଉଁ ମୁଖ୍ୟ ପ୍ରତିବନ୍ଧକକୁ ଆପଣ ସାମ୍ନା କରିଥିଲେ ?

7. How do you think the Committee could do more? What else is needed?
କମିଟି ଆଉ କିଛି କରିପାରିବ ବୋଲି ଆପଣ ଭାବୁଛନ୍ତି କି ଏଥି ପାଇଁ ଆଉ କଣ ଆବଶ୍ୟକ ରହିଛି ?
