# Supplementary material for: The challenges of institutionalizing community-level social accountability mechanisms for health and nutrition: a qualitative study in Odisha, India
Source: BMC Health Serv Res. 2018 Oct 19;18:788. doi: 10.1186/s12913-018-3600-1 (PMC6194642; doi:10.1186/s12913-018-3600-1)
Supplement: Supplementary file 4 — In-Depth Interview Schedule – Committee Members. (PDF 497 kb) [file 12913_2018_3600_MOESM4_ESM.pdf]

DRAFT - IDI with Committee members

Interview code:

Audio File code:

Date:

District: Block:

Village: Location of interview:

Interviewer Name: Note Taker Names:

CONSENT OF RESPONDENT

ଉତ୍ତରଦାତାଙ୍କ ସମ୍ମତି ପ୍ରଦାନ

Thank you for this opportunity to speak with you. Together with the Institute for Development Studies (IDS), we are conducting a survey that will provide us with necessary information to carry out research that is designed to help promote the welfare of people in Odisha; particularly, to improve food consumption and nutrition of the people, and to enhance community accountability and income generation.

ଆପଣଙ୍କ ସହିତ ଆଲୋଚନା କରିବା ପାଇଁ ସୁଯୋଗ ମିଳିଥିବାରୁ ଧନ୍ୟବାଦ ଜଣାଉଛି । ଆମେ institute of development studies (IDS) ସହିତ ଏକ ସର୍ଭେ କାର୍ଯ୍ୟ କରୁଛୁ ଏଥିରୁ ଯେଉଁ ସୁଚନା ସବୁ ମିଳିବ ତାହାକୁ ଆଧାର କରି ଆମ୍ଭେ ଏକ ଅନୁଧ୍ଯାନ ବା research କରିବୁ ଯେଉଁଥିରେ କି ଓଡିଶା ବାସିଙ୍କ ମଗଂଳ ବିଶେଷ କରି ସମାନକଂର ଖାଦ୍ୟା ଖାଇବା ଏବଂ ଲୋକମାନକଂର ପୌଷ୍ଟିକ ସ୍ଥିତି ତଥା ଆୟ ପନ୍ଥା ସୃଷ୍ଟି ଏବଂ ଗୋଷ୍ଠି ଉତ୍ତର ଦାଇତ୍ଵ ସ୍ଥାନ ପାଇବ ।

We are inviting you to be a participant in this study. We value your opinion and there are no wrong answers to the questions we will be asking in the interview. We will use approximately 45 minutes of your time to collect all the information. There will be no cost to you other than your time. There will be no risk as a result of your participating in the study. Your participation in this research is completely voluntary. You are free to withdraw your consent and discontinue participation in this study at any time.

This study is conducted anonymously. You will only be identified through code numbers. Your identity will not be stored with other information we collect about you. Any information we obtain from you during the research will be kept strictly confidential. This interview will be audio recorded and its content will not be shared or used outside the scope of this research.

ଏହି ଅନୁଧ୍ଯାନରେ ଏକ ଅଂଶ ଗ୍ରହଣ କରି ହେବା ପାଇଁ ଆମେ ଆପଣଙ୍କୁ ଅନୁରୋଧ କରୁଛୁ ଆମେ ପଚାରିବା ଯେ କୌଣସି ପ୍ରଶ୍ନ ପାଇଁ ଆପଣଙ୍କ ମତାମତ କୁ ଆମେ ଗୁରତ୍ଵର ସହ ଗ୍ରହଣ କରିବୁ ଆଉ ଏକ କଥା କେଉଛି ଏଥିରେ କୌଣସି ଉତ୍ତର ଭୁଲ୍ ଠିକ୍ ର ବିବେଚନା ମଧ୍ୟ କରଜିବ ନାହିଁ । କଥାଟି ହେଲା ଆପଣ କେବଳ ଏଥିପାଇଁ ସମୟ ଦେବା ବ୍ୟତିତ ଆପଣଙ୍କୁ ଆଉ କିଛି ଦବାର ଆବସ୍ୟାକତା ନାହିଁ । ସୁଚନା ସଂଗ୍ରହ ନିମନ୍ତେ ଆମ୍ଭେ ଆପଣଙ୍କର ୪୫ ମିନିଟ୍ ସମୟ ନେବୁ ଏହି ଅନୁଧ୍ଯାନର ଆପଣଙ୍କ ଅଂଶ ଗ୍ରହଣ ସମ୍ପୁର୍ଣ ଇଛାଧିନ । ଏହି ଅନୁଧ୍ଯାନରେ ଅଂଶ ଗ୍ରହଣ କରିବାର ସମ୍ମତି କୁ ଆପଣ ଯେ କୌଣସି ସମୟରେ ଫେରାଇ ଦେଇ ଅନୁଧ୍ଯାନ ଅଂଶ ଗ୍ରହଣ କରିବୁ ମନା କରିଦେଇ ପାରନ୍ତି । ଅନୁଧ୍ଯାନ ଟିରେ ସମ୍ପୁର୍ଣ ଗୋପନୀୟତା ଭାଷା କରା ଯାଇଛି ଏଥିରେ ଆପଣଙ୍କ ପରିଚୟ ଏକ କୋଡ୍ ନମ୍ବର୍ ହିଁ ରହିବ ଅନ୍ୟ କୌଣସି ସୁଚନା ସହିତ ଆପଣଙ୍କ ପରିଚୟ କୁ ସଲଗ୍ନ କରାଯିବ ନାହିଁ ଅନୁଧ୍ଯାନ ପ୍ରକ୍ରିୟା ରେ ଆପଣଙ୍କ ଠାରୁ ସମ୍ପୁର୍ଣ ଗୋପନ ରଖାଯିବ । ଏହି ସାକ୍ଷାତକାରଟିକୁ ଆମେ ରେକର୍ଡ କରିବାକୁ ଚାହୁଁଚୁ ଏହାକୁ କେବଳ ଆମେ ରିସର୍ଚ୍ ପାଇଁ ବ୍ୟବହାର କରିବୁ , ଅନ୍ୟ କେଉଁଠି ପ୍ରକାଶ କରାଯିବନାହି.

Your participation will be highly appreciated. The answers you give will help provide better information to policy-makers, practitioners and program managers so that they can plan for better services that will respond to your needs.

ଅନୁଧ୍ଯାନରେ ଆପଣଙ୍କ ଅଂସ ଗ୍ରହଣ ପ୍ରଶଂଶନିୟ ହୋଇ ରହିବ । ଅନୁଧ୍ଯାନ ମାଧ୍ୟମରେ ଆପଣଙ୍କ ଉତ୍ତର ଓ ସୁଚନା ଗୁଡିକ ନୀତି ନିୟମ ପ୍ରସ୍ତୁତ କରି, କାର୍ଯ୍ୟକାରୀ ପେଶାଦାର, କାର୍ଯ୍ୟକ୍ରମ ପରିଚାଳନା କାରିକୁ ଖୁରାକ ଯୋଗାଇବା, ଫଳତଃ ସେମାନେ ଆପଣଙ୍କ ଉତ୍ତର ମାଧ୍ୟମରେ ଉପସ୍ଥାପନ କରିଥିବା ଆବଶ୍ୟକତାର ପୁରଣ ନିମନ୍ତେ ଓ ସେବା ଯୋଗାଣ ନିମନ୍ତେ ଉତ୍ତମ ଯୋଜନା ପ୍ରସ୍ତୁତ କରିବାରେ ଉତ୍ତମ ଯୋଜନା ଅରିପାରିବେ ।

The researcher read to me orally the consent form and explained to me its meaning. I agree to take part in this research. I understand that I am free to discontinue participation at any time if I so choose, and that the investigator will gladly answer any question that arise during the course of the research.

ଅନୁଧ୍ୟାନକାରି ସମ୍ମତି ପତ୍ର କୁ ମୋ ସାମ୍ନାରେ ସମ୍ପୁର୍ଣ ଭାବେ ପଢ଼ି ସଣାଇ ଛନ୍ତି ଏବଂ ଏହାର ଅର୍ଥ ମତେ ବୁଝାଇ ଛନ୍ତି ଏହି ଅନୁଧ୍ୟାନ ରେ ଭାଗ ନେବା ପାଇଁ ମୁ ରାଜି । ମୁ ଭଲଭାବେ ଜନିଛି ଯେ ଯେକୌଣସି ସମୟରେ ମୁ ଅନୁଧ୍ୟାନର ନିଜକୁ ହେରାଇ ଆଣିପାରିବ ଏବଂ ଅନୁଧ୍ୟାନକାରି ଇଣ୍ଟରଭ୍ୟୁ ମଧ୍ୟରେ ଉପୁଜିଥିବା ପ୍ରଶ୍ନ ଗୁଡିକ ର ଉତ୍ତର ଖୁସିରେ ଦେବେ ।

Contact Persons:

ଯୋଗାଯୋଗ ଠିକଣା

Satyanarayan Mohanty, DCOR Consulting

Address: DCOR Consulting Pvt. Ltd., 131 (P), Punjabi Chhak, Satyanagar, Odisha, India, Pin – 751007

Tel: +91-9437698965, E-mail: satya.dcor@gmail.com

Dr. Nicholas Nisbett, IDS

Address: Institute of Development Studies, University of Sussex, Brighton BN1 9RE

Tel: +44 (0)1273 606261; E-mail: n.nisbett@ids.ac.uk

Please tick mark on the right box depending on the respondent’s consent

ଉତ୍ତର ଦାତା/ଦାତ୍ରୀ ସମ୍ମତିକୁ ଭିତି କରି ନିରଧିଷ୍ଟ କୋଠରୀରେ ଠିକ୍ ଚିହ୍ନ ଦିଅନ୍ତୁ

Consent given: ସମ୍ମତି ପ୍ରଦାନ	Yes	No

Signature of the Enumerator: ______________________   **Date: DD/_______/_______/_______/**

A. Preliminary Information: ମୌଳିକ ସୂଚନା

1. Age: ବୟସ

2. Gender: ଲିଂଗ

3.Years/months in service in this position: ଉକ୍ତ ପଦବୀରେ କେତେ ମାସ/ବର୍ଷ ହେଲା ସେବାଦେଇ ଅସୁଛନ୍ତି

4.Cate/Ethnicity:ଜାତି/ପାରମ୍ପରିକ ବର୍ଗ

5. Religion:

6. Education: ଶିକ୍ଷା

7. Occupation (Previous occupation if retired): ବେଉଶା (ଯଦି ଅବସର ପ୍ରାପ୍ତ ତେବେ ପୁର୍ବରୁ ଥିବା ବେଉଶା ବ ବୃତି)

8. Committee(s) of the interviewee: ସାକ୍ଷାତକାର ଦେଉଥିବା ବ୍ୟକ୍ତି କେଉଁ କମିଟିର?
A. Name of Committee: କମିଟିର ନାମ ___________________________ Position held: କେଉଁ ପଦବୀରେ ଅଛନ୍ତି?_____________________ Years/months in the position: ___________ଉକ୍ ପଦବୀରେ କେତେ ମାସ/ବର୍ସ ହେବ ଅଛନ୍ତି?

B. Name of Committee: କମିଟିର ନାମ ___________________________ Position held: କେଉଁ ପଦବୀରେ ଅଛନ୍ତି?_____________________ Years/months in the position: ଉକ୍ ପଦବୀରେ କେତେ ମାସ/ବର୍ସ ହେବ ଅଛନ୍ତି?____________________

C. Name of Committee: କମିଟିର ନାମ ___________________________ Position held: କେଉଁ ପଦବୀରେ ଅଛନ୍ତି?_____________________ Years/months in the position: ଉକ୍ ପଦବୀରେ କେତେ ମାସ/ବର୍ସ ହେବ ଅଛନ୍ତି?__________

D. Other affiliations: ଅନ୍ୟାନ୍ୟ ସମ୍ପୃକ୍ତି

____

Note: Include affiliations with PRI, Government agencies, CBOs and NGOs. ଦ୍ରଷ୍ଟବ୍ୟ: ଏଥି ମଧରେ ପନଚୟଟି ରାଜ ଅନୁଷ୍ଠାନ, ସରକାରୀ ସଂସ୍ଥା,ଗୋଷ୍ଠି ଭିତ୍ତିକ ଅନୁସ୍ଥାନ ଏବଂ ସ୍ବେଚ୍ଛାସେବୀ ଅନୁସ୍ଥାନ ଅନ୍ତର୍ଭୁକ୍ତ.

B. Committee role and effectiveness କମିଟିର ଭୁମିକା ଏବଂ ଫଳପ୍ରଦତ୍ତା

1. Could you tell us how you became involved in the Committee? ଆପଣ କମିଟିରେ କିପରି ସାମିଲ୍ ହେଲେ ତାହା କହିବେ କି?
Prompts: What was the motivation behind it? ସୁଚାଇ ପଚାରନ୍ତୁ: ଅଥିରେ ସାମିଲ୍ ହେବ ପାଇଁ ଆପଣ କି ପ୍ରକାରର ପ୍ରୋସ୍ଥାହନ ପାଇଥିଲେ?
How were you appointed? ଆପଣକିପରି ନିଯୁକ୍ତି ପାଇଲେ?

2. Could you please describe some of the activity the Committee does in your village?

କମିଟି ଆପଣଙ୍କ ଗାଁ ରେ କରୁଥିବା କେତେକ କାମ ବିଷୟରେ ଆମକୁ ବୁଝାଇ କହିବେକୀ?

Prompts: Who is involved in these activities? Who benefits from them?
ସୁଚାଇ ପଚାରନ୍ତୁ: ଏହି କାମ ଗୁଡିକରେ କେଉଁମାନେ ସାମିଲ୍ ଥାନ୍ତି? କେଉଁ ମନେ ଏଥିରେ ଉପକୃତ ହୁଅନ୍ତି?

3. What is your role in the Committee?
କମିଟିରେ ଆପଣକର ଭୁମିକ ସବୁ କଣ?

(Note: what do you *actually* do in the Committee)
(ଦ୍ରଷ୍ଟବ୍ୟ: କମିଟିରେ ଆପଣ ପକୃତପକ୍ଷେ କେଉଁ କାମ କରିଥାନ୍ତି?)

4. How do these activities matter to you? Why are they important?
ଏହିକାମ ଗୁଡିକରେ ଆପଣକର ସମ୍ପୃକ୍ତି କଣ? ଏଥିରୁ ଆପଣ କଣ ପାଇଥାନ୍ତି?

ଏଗୁଡିକ କାହିଁକି ଗୁରୁତ୍ୟପୁର୍ଣ?

5. Could you tell us about an achievement the Committee obtained? What was the achievement? And how do you think was that obtained?
କମିଟି କେଉଁ ସବୁ ସଫଳତା ଅର୍ଜନ କରିଛି କହିବେକି? ସଫଳତା ଗୁଡିକ କଣ? ସେହି ସଫଳତା କିପରି ମିଳିଥିଲା ବୋଲି ଆପଣ ଭାବୁଛନ୍ତି?

6. Are there activities the Committee cannot complete? Why is it so? What are the challenges in executing some of these activities?
ଏପରି କିଛି ଅଛି କାମ ଯାହା କମିଟି ସପୁର୍ଣ କରିପାରିନି? ଏପରି କାହନିଂକି ହେଲା? ଏହି କାମ ଗୁଡିକ କରିବାରେ କେଉଁ ସବୁ ବାଧା ଉପୁଜିଥିଲା?

<u>C. Participation and decision-making ଅଂଶଗ୍ରହଣ ଏବଂ ନିସ୍ପତି ଗ୍ରହଣ!</u>

1. Who are the other members of the Committee? How were they appointed?
କମିଟିରେ ଆଉ କେଉଁ ମନେ ସଦସ୍ୟ/ସଦସ୍ୟା ଅଛନ୍ତି? ସେମାନେ କିପରି ଏଥିରେ ନିଯୁକ୍ତି ପାଇଥିଲେ?

2. Could you talk about the last meeting you attended?
Prompts: Who convened the meeting, how often are they usually held and how often do you usually attend?
ଆପଣ ଉପସ୍ତିତ ଥିବା ଗତ କମିଟି ବୈଠକ ବାବଦରେ କିଛି କହିବେକୀ?

ସୁଚାଇ କୁହନ୍ତୁ:ବୈଠକରେ କିଏ ଅଧକ୍ଷତା କରିଥିଲେ? କେତେଦିନରେ ଥରେ (ବ୍ୟବଧାନରେ) ଏହି ବୈଠକ ହୋଇଥାଏ ଏବଂ ଆପଣ କେବେ କେବେ ଏଥିରେ ଯୋଗ ଦିଅନ୍ତି?

(If was not answered previously: What did you discuss during the last meeting? And what was the outcome of the discussion?)
(ଯଦି ପୁର୍ବ ପ୍ରଶ୍ନ ର ଉତ୍ତର ଦେଇ ନଥିବେ)ଗତ ବୈଠକରେ ଆପଣ କଣ ସବୁ ଆଲୋଚନା କରିଥିଲେ? ଏହି ଆଲୋଚନା ର ଫଳାଫଳ କଣଥିଲା କହିବେକି?

3. During meetings, do you feel comfortable in speaking up or taking part in the discussions? If not, why is it so?
ବୈଠକରେ କହିବାକୁ ଏବଂ ଭାଗନେବାକୁ ଆପଣକୁ ଭଲଲଗେ କି? ଯଦି ଭଲ ଲଗେନି ତେବେ କଣପାଇଁ ଭଲ ଲଗେନି?
Prompts: Who chairs the meeting? Why do you think some people don't participate?
ସୁଚାଇ କୁହନ୍ତୁ:ବୈଠକରେ କିଏ ସଭାପତିତ୍ୱ କରନ୍ତି? କିଛି ଲୋକ ଏଥିରେ ଅଂଶଗ୍ରହଣ କରନ୍ତିନାହିଁ ବୋଲି ଆପଣ କାହିଁକି ଭାବୁଛନ୍ତି?

54. What do you think are the barriers members face in a) organizing meetings b) attending meetings and c) actively participating in meetings?
୧)ସଦସ୍ୟା/ସଦସ୍ୟମାନେ ବୈଠକରେ ଯୋଗଦେବାରେ ୨) ବୈଠକ ଆୟୋଜନ କରିବାରେ ୩)ବୈଠକରେ ସକ୍ରିୟ ଅଂଶଗ୍ରହଣ କରିବାରେ ସମ୍ମୁଖିନ ହେଉଥିବା ପ୍ରତିବନ୍ଧକ ବାବଦରେ ଆପଣ କଣ ଭାବନ୍ତି?
(Note: enquiry about non-tangible barriers such as caste and gender representation and social status of committee members)
(ଦ୍ରଷ୍ଟବ୍ୟ: ଦେଖାଯାଉନଥିବା ପ୍ରତିବନ୍ଧକ ଯଥା ଜାତି ଏବଂ ଲିଂଗ ଭେଦ ଜନିତ ଓ କମିଟି ସଦସ୍ୟ ଓ ସଦସ୍ୟଙ୍କ ସମାଜିକସ୍ତିତି ବୈଷଇକ ପ୍ରତିବନ୍ଧକ ବାବଦରେ ପଚାରି ବୁଝନ୍ତୁ?

5. If you had to raise an issue with the activities of the Committee, how would you go about it? Who would you approach and how? (Note: if possible/relevant, make examples from past experience)
କମିଟିର କୌଣସି କାର୍ଯ୍ୟ କଳାପ ସଂକ୍ରାନ୍ତିୟ ସମସ୍ୟା ବାବଦରେ କହିବାକୁ ଚାହିଁଲେ ଏହାକୁ କିପରି ଉପସ୍ଥାନ କରିଥାନ୍ତି ? ଆପଣ ଏବାବଦରେ କାହାକୁ କିପରି ଭାବେ କହିଥାନ୍ତି ?

D. Community Mobilization on health and nutrition(ସ୍ୱାସ୍ଥ୍ୟ ଓ ପୃସ୍ଟିରେ ଗୋସ୍ଠୀ ସଂଚାଳନ)

1. What do you think are some of the main issues women, especially mothers, in your village face?
ଆପଣଙ୍କ ଗ୍ରାମରେ ମହିଳାମାନେ ବିଶେଷକରି ମାମାନେ କେଉଁ ସବୁ ମୁଖ୍ୟ ସମଶ୍ୟା ର ସମ୍ମୁଖିନ ହୋଇଥାନ୍ତି?

2. What do you feel is the root cause for these issues?
ସମସ୍ୟା ର ମୂଳକାରଣ କଣ ବୋଲି ଆପଣ ଭାବୁଛନ୍ତି?
Answer, then prompt: do you feel some groups of women face particular challenges? Who (SC,ST etc) and how?
ଉତ୍ତର ସୂଚାଇ କୁହନ୍ତୁ: କିଛି କମିଟିର ମହିଳା ମନେ ସ୍ୱତନ୍ତ ସମସ୍ୟାର ସମ୍ମୁଖିନ ହୋଇଛନ୍ତିକି? ଯେକି (ତଫସିଲି ଭୁକ୍ତ ଜାତି, ଜନଜାତି.ଇତ୍ୟଦି) କିପରି ସମ୍ମୁଖିନ ହୁଅନ୍ତି?

3. Do you feel these Committees have a role in addressing them? And if so, how?
ଆପଣ ଭାବୁଛନ୍ତିକି ଏହି ସମସ୍ୟାର ସମାଧାନରେ କମିଟିର କିଛି ଭୁମିକା ରହିଛିକି? ଏବଂ ଯଦି ଅଛି ଏହା କିପରି?

4.What do you feel is the impact of the Committee in promoting women's health?
ମହିଳା ଙ୍କ ସ୍ୱାସ୍ଥ୍ୟ ଅବସ୍ଥାରେ ଉନ୍ନତି ଆଣିବାରେ କମିଟିର ପ୍ରଭାବ ପଡିଛି ବୋଲି ଆପଣ ଭାବୁଛନ୍ତି କି ?

Prompts: For instance, how is improved your status within the community? Or how has it improved access to food/health services, knowledge of entitlements/service delivery

ସୁଚାଇ କୁହନ୍ତୁ: ଉଦାହରଣ ସ୍ବରୁପ ଗୋଷ୍ଠୀରେ ଆପଣଂକ ଅବସ୍ଥାରେ କିପରି ଉନ୍ନତି ଘଟିଛି? ବା ଏହାଦ୍ବାରା ଖାଦ୍ୟ/ସ୍ବାସ୍ଥ୍ୟସେବା ପ୍ରଦାନ/ଅଧିକାର ବିସୟକ ଜ୍ଞାନ ରେ ଉନ୍ନତି କିପରି ଘଟିଛି?

5. How does the committee engage with pregnant women and mothers of young children?
କମିଟି ଗର୍ଭବତୀ ଏବଂ ବଢାନ୍ତା ଶିଶୁ ଙ୍କ ମା ମାନଙ୍କ ସହିତ କିପରି ଚର୍ଚ୍ଚା କରନ୍ତି?

Prompts: What do you tell women when you approach them.
ମହିଳା ମାନଂକୁ କଣ କହି ବୁଝେଇଥାନ୍ତି.?
What type of communication does the committee have?
କମିଟି କେଉଁ ପ୍ରକାରରେ ବୁଝାଇଥାନ୍ତି?

What are the main challenges you encounter?
ଆପଣ କେଉଁ ସବୁ ମୁଖ୍ୟ ପ୍ରତିବନ୍ଧକର ସମୁଖିନ ହୋଇଥିଲେ?

6. How do you think the Committee could be more? What else is needed?
କମିଟି ଆଉ କିଛି ଅଧିକ କରିପାରିବ ବୋଲି ଆପଣ ଭାବୁଛନ୍ତି କି ?ଏଥି ପାଇଁ ଆଉ କଣ ଆବଶ୍ୟକ ରହିଛି ?

E. Funding (applies only to GKS) ପାଣ୍ଠି ଯୋଗାଣ (କେବଳ ଗାଁ କଲ୍ୟାଣ ସମିତି ନିମନ୍ତେ)

1. Has the Committee you are part of received any funding in the last two years?
ଗତ ଦୁଇ ବର୍ଷ ମଧ୍ୟରେ ଆପଣ ରହିଥିବା କମିଟି କିଛି ଅନୁଦାନ ପାଣ୍ଠି ପାଇଛିକି?

2. If so, how was it spent?
ଯଦି କିଛି ପାଇଛି, ଏହି ପାଣ୍ଠି କିପରି ଖର୍ଚ୍ଚ ହୋଇଥିଲା?
Prompt: How was that decision taken and by whom?
ସୁଚାଇ କୁହନ୍ତୁ: ଏହି ନିସ୍ବତି କିପରି ଏବଂ କାହଦ୍ବାରା ନିଆଯାଇଥିଲା?
3. What were the factors considered for this decision?
ଏହି ନିଷ୍ପତି ନେବା ପାଇଁ କେଉଁ ବାବଦରେ ବିଚାର କରିଥିଲେ ?

4. How has the expenditure been documented or recorded? (Observation) (ନିରକ୍ଷଣ କରିବା)
ଖର୍ଚ୍ଚର ବିବରଣୀ ଓ ହିସାବକିତାବ କିପରି ଭାବେ ଟିପି ରଖାଯାଇଛି?
